# SOX9 reprograms endothelial cells by altering the chromatin landscape

**DOI:** 10.1093/nar/gkac652

**Published:** 2022-07-29

**Authors:** Bettina M Fuglerud, Sibyl Drissler, Jeremy Lotto, Tabea L Stephan, Avinash Thakur, Rebecca Cullum, Pamela A Hoodless

**Affiliations:** Terry Fox Laboratory, BC Cancer, Vancouver, British Columbia V5Z 1L3, Canada; Department of Medical Genetics, University of British Columbia, Vancouver, British Columbia V6H 3N1, Canada; Department of Biosciences, University of Oslo, 0316 Oslo, Norway; Terry Fox Laboratory, BC Cancer, Vancouver, British Columbia V5Z 1L3, Canada; Cell and Developmental Biology Program, University of British Columbia V6T 1Z3, Vancouver, British Columbia, Canada; Terry Fox Laboratory, BC Cancer, Vancouver, British Columbia V5Z 1L3, Canada; Cell and Developmental Biology Program, University of British Columbia V6T 1Z3, Vancouver, British Columbia, Canada; Terry Fox Laboratory, BC Cancer, Vancouver, British Columbia V5Z 1L3, Canada; Cell and Developmental Biology Program, University of British Columbia V6T 1Z3, Vancouver, British Columbia, Canada; Terry Fox Laboratory, BC Cancer, Vancouver, British Columbia V5Z 1L3, Canada; Department of Medical Genetics, University of British Columbia, Vancouver, British Columbia V6H 3N1, Canada; Terry Fox Laboratory, BC Cancer, Vancouver, British Columbia V5Z 1L3, Canada; Terry Fox Laboratory, BC Cancer, Vancouver, British Columbia V5Z 1L3, Canada; Department of Medical Genetics, University of British Columbia, Vancouver, British Columbia V6H 3N1, Canada; Cell and Developmental Biology Program, University of British Columbia V6T 1Z3, Vancouver, British Columbia, Canada; School of Biomedical Engineering, University of British Columbia, Vancouver, British Columbia V6T 1Z3, Canada

## Abstract

The transcription factor SOX9 is activated at the onset of endothelial-to-mesenchymal transition (EndMT) during embryonic development and in pathological conditions. Its roles in regulating these processes, however, are not clear. Using human umbilical vein endothelial cells (HUVECs) as an EndMT model, we show that SOX9 expression alone is sufficient to activate mesenchymal genes and steer endothelial cells towards a mesenchymal fate. By genome-wide mapping of the chromatin landscape, we show that SOX9 displays features of a pioneer transcription factor, such as opening of chromatin and leading to deposition of active histone modifications at silent chromatin regions, guided by SOX dimer motifs and H2A.Z enrichment. We further observe highly transient and dynamic SOX9 binding, possibly promoted through its eviction by histone phosphorylation. However, while SOX9 binding is dynamic, changes in the chromatin landscape and cell fate induced by SOX9 are persistent. Finally, our analysis of single-cell chromatin accessibility indicates that SOX9 opens chromatin to drive EndMT in atherosclerotic lesions *in vivo*. This study provides new insight into key molecular functions of SOX9 and mechanisms of EndMT and highlights the crucial developmental role of SOX9 and relevance to human disease.

## Graphical Abstract

**Graphical Abstract:**
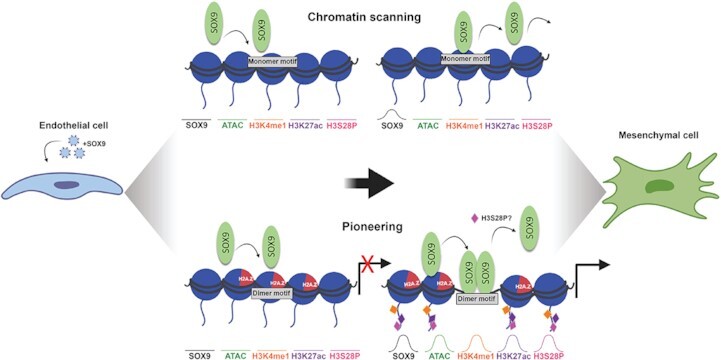
SOX9 binds closed chromatin by transient scanning or by specific binding to regions containing dimer motifs and H2A.Z. In the latter, SOX9 opens chromatin and enriches for active histone modifications.

## INTRODUCTION

Around 20 SOX transcription factors (TFs) with divergent developmental functions have been identified, of which many act as master regulators that control critical transcriptional programs to determine cell fate and differentiation (1). SOX9, a member of the SOXE family of SOX factors, contains a highly conserved high mobility group (HMG) domain binding to ACAAa/tG-like DNA sequences. The HMG domain was first identified in *SRY*, a crucial factor for mammalian sex determination (2,3). SOXE factors (SOX8, SOX9 and SOX10) also possess a dimerization domain upstream of the HMG domain that mediates DNA-dependent dimerization to a palindromic ACAAa/tGnnnnCa/tTTGT DNA sequence (4,5). Mutations in and around the *SOX9* gene, some that specifically disrupt dimerization, are known to cause Campomelic Dysplasia (CD), a skeletal dysmorphology syndrome characterized by bowing of the limb long bones, male-to-female sex reversal and a variety of congenital heart defects (6,7).

Most of our understanding regarding biological functions of SOX9 has come from studies involving chondrogenesis, which have revealed its role in regulating extracellular matrix (ECM) production and organization (8,9). SOX9 is also crucial for processes involving epithelial-to-mesenchymal transition (EMT) and the closely related process endothelial-to-mesenchymal transition (EndMT) (10–12). SOX9 can drive cancer invasion and metastasis in multiple cancers, including prostate, breast, colon, gastric, and lung (summarized in (13)) and is involved in promoting liver and cardiac fibrosis by stimulating ECM deposition (14,15). Recently, SOX9 expression in endothelial cells was also found to induce skin wound healing (16). During cardiac valve development, SOX9 is essential during cushion formation, which involves EndMT, and where SOX9 activates transcription of key regulators of heart valve development (11,17). Although several lines of evidence indicate that SOX9 is essential for promoting a mesenchymal phenotype, the mechanisms of SOX9 in EndMT induction have not been evident.

At the molecular level, SOX9 has been suggested to alter epigenetic signatures, but most of the work has been done on a single locus in the *COL2A1* enhancer. SOX9 remodeled chromatin and activated transcription at this site on an *in vitro* assembled chromatin template (18). In the same study, a CD mutation which affects SOX9 dimerization was shown to impair chromatin remodeling and transcriptional activation by SOX9. In chondrogenic systems, SOX9 can activate enhancers (19), but may not be required for the initiation of chromatin changes (20).

Some developmental TFs have the remarkable ability to reprogram one cell type into another by engaging genes that are developmentally silent and in closed chromatin, thus acting as pioneer TFs to initiate transcriptional events (21). In hair follicle stem cells, SOX9 has been put forward as a pioneer TF (22); however, while this study showed increased activation of enhancers, it did not demonstrate whether SOX9 opens chromatin, a hallmark of pioneer TFs. SOX9 acts as a master regulator in heart valve development, binding regulatory regions that control TFs important for proper valve development, but its impact on the chromatin landscape is not yet characterized (17). In summary, the epigenetic requirements of SOX9 to alter chromatin and drive cell fate decisions remains uncertain and may vary between cell types.

Here, we have used human umbilical vein endothelial cells (HUVECs) to study cell reprogramming initiated by SOX9 and its role in restructuring the chromatin landscape. Altogether, we find that at a subset of regions that are silent in unstimulated HUVECs, SOX9 occupancy increases chromatin accessibility and enrichment of active histone modifications to induce expression of mesenchymal genes, suggesting SOX9 has the ability to act as a pioneer TF to reprogram cell fate. This function is motif encoded and occurs predominantly in distal regulatory regions with enrichment of the histone variant H2A.Z. Furthermore, we show that SOX9 chromatin binding is highly dynamic, but nevertheless causes stable changes in the chromatin landscape. Using *in vitro* and *in vivo* methods, we show that H3S28 phosphorylation prevents SOX9 histone binding suggesting H3S28 phosphorylation might be involved in regulating SOX9 chromatin binding dynamics. Together, our work demonstrates how a single TF can have widespread effects on chromatin structure and reprogram cell fate, both in normal embryonic development as well as in pathogenesis.

## MATERIALS AND METHODS

### Mice

CD1 Elite mice (Strain Code: 482, Charles River) were maintained in accordance with the University of British Columbia's Animal Care Committee's standards under specific pathogen-free conditions on a 12-h light-dark cycle. Embryos were collected in ice-cold phosphate-buffered saline (PBS) under a Leica MZ6 dissecting scope. Sex-specific differences were not anticipated and, as such, embryo sex was not determined.

### Cell culture

HUVECs (C0035C, Thermo Fisher Scientific) were maintained in Medium 200 supplemented with 2% low serum growth supplement (Thermo Fisher Scientific), with penicillin and streptomycin. HUVECs were plated in six-well plates and lentiviral supernatants were titrated to transduce >95% of the cells (indicated by GFP expression). Media was replaced 24 h after transduction and cells were collected 72 h later or seeded for migration assays or PLA. SOX9 overexpression was confirmed by western blotting and RT-qPCR (described in Supplemental Material).

### Immunostaining

Hearts were micro-dissected from E9.5, E10.5 and E12.5 embryos, washed with PBS, and fixed with 4% paraformaldehyde (PFA) at 4°C for 4–12 h. Ventricles were punctured with fine dissecting forceps prior to fixation at E10.5 and E12.5. Embryos were washed with PBS three times, transferred through a sucrose gradient (15%–30%–60% sucrose in PBS, 4°C, 1–12 h each) and embedded in TissueTek optimal cutting temperature compound. Tissues were frozen on dry-ice and stored at –80°C. 8 μm sections were collected using a Leica CM3050S cryostat at –25°C with SuperFrost Plus slides. Sections were circumscribed with an Elite PAP pen (Diagnostic Biosystems K039) and slides were placed in an opaque humidity chamber. Sections were subsequently re-fixed, blocked, stained, and mounted as described for HUVECs. For HUVEC immunostaining, cells were washed with PBS and fixed with 4% PFA for 10 min at room temperature (RT). After washing in PBS, the cells were incubated in block solution (5% bovine serum albumin, 0.1% Triton-X in PBS) for 1 h at RT, incubated with primary antibodies in block solution overnight at 4 ºC, washed in PBS, and incubated with species-specific Alexa Fluor 568-, 594- or 647-conjugated secondary antibodies in block solution (all at 1:500) for 1 h at RT. After washing in PBS, cells were counterstained with 1:1000 DAPI (Thermo Fisher Scientific), washed again with PBS, and mounted using a minimal volume of 25 mg/ml DABCO (Sigma) in 9:1 glycerol:PBS. The cells were then mounted using a minimal volume of 25 mg/ml DABCO (Sigma) in 9:1 glycerol:PBS. Images were captured using a Nikon Instruments Eclipse Ti confocal laser microscope and image capture and processing was done using Fiji in ImageJ (23) with brightness, contrast, and pseudo-coloring adjustments applied equally across all images in a given series. Antibodies used were: goat anti-SOX9 (1:50, AF3045, R&D Systems), rabbit anti-ERG (1:50, ab92513, Abcam), rabbit anti-VIM (1:50, 5741S, Cell Signaling), rabbit anti-POSTN (1:50, ab14041, Abcam), rat anti-PECAM1 (1:50, 102501, Biolegend), donkey anti-goat IgG, A568 (A-11057, Thermo Fisher Scientific), donkey anti-rabbit IgG, A568 (A-10042, Thermo Fisher Scientific), Donkey anti-rat IgG, A594 (A-21209), and donkey anti-rabbit IgG, A647 (A-31573, Thermo Fisher Scientific).

### Migration assays

Transwell invasion assays were carried out according to manufacturer's recommendation. Briefly, 15 000 HUVECs were seeded in the upper chamber of 5 μm 24-well polycarbonate transwell inserts (Corning). HUVECs were maintained for 24 h under normal culture conditions. After 24 h, the non-migratory cells were removed from the upper chamber with a cotton swab. The membrane was washed with PBS, fixed with 4% PFA for 10 min and stained with DAPI nuclear stain for 10 min. The lower side of the membrane was photographed with a Zeiss AxioImager fluorescence microscope. Cells that had migrated through the membrane were counted using Fiji ImageJ (23). To control for cell death and proliferation, transduced HUVECs of equal density were monitored for differences after 24 h.

### RNA-seq and analysis

RNA was extracted using TRIzol (Thermo Fisher Scientific). Three biological replicates were sequenced. Reads were aligned to Hg19/ GRCh37 using STAR (24). Fragments per kb of exon per million reads (FPKMs) were calculated using Cufflinks (25). Differential expression was determined by log2 ratio between SOX9 overexpression and empty vector FPKM values. For downstream analyses, only genes with log_2_ ratio >0.5 or <–0.5 were included (*P*-value < 0.05). To determine hallmark gene sets and cell type signature gene sets we used GSEA (26).

### CUT&RUN

CUT&RUN was performed as described (27). Briefly, 500 000 cells were washed with Wash Buffer (20 mM HEPES pH 7.5, 150 mM NaCl, 0.5 mM spermidine and protease inhibitors), bound to Concanavalin A-coated magnetic beads (Bangs Laboratories) and incubated with SOX9 antibody (1:100, AB5535, Millipore) diluted in wash buffer containing 0.05% digitonin (Dig-Wash) overnight at 4°C. Cells were washed and incubated with Guinea Pig anti-Rabbit secondary antibody (1:100, Novus Biologicals) diluted in Dig-Wash for 1 h at RT, washed again and incubated with CUTANA™ pAG-MNase (Epicypher) for 10 min at RT. Slurry was washed, placed on ice and incubated with Dig-Wash containing 2 mM CaCl_2_ for 30 min to activate digestion. Stop buffer (340 mM NaCl, 20 mM EDTA, 4 mM EGTA, 0.05% Digitonin, 0.05 mg/ml glycogen, 5 μg/ml RNase A) was added, and fragments were released by 30 min incubation at 37°C. DNA was extracted with phenol-chloroform and ethanol precipitation. Libraries were constructed and sequenced. Two biological replicates of SOX9 at 96 h and one replicate of the SOX9 time-course were sequenced.

### CUT&Tag

CUT&Tag was performed as described (28). Cells were washed, and incubated with primary and secondary antibodies as described for CUT&RUN. Cells were washed again and incubated with pA-Tn5 adapter complex diluted in Dig-300 buffer (0.01% Digitonin, 20 mM HEPES, pH 7.5, 300 mM NaCl, 0.5 mM Spermidine, protease inhibitors) for 1 h at RT, then washed and incubated with Dig-300 buffer containing 10 mM MgCl_2_ for 1 h at 37°C. To stop tagmentation, 16.6 mM EDTA, 0.1% SDS, and 50 μg Proteinase K was added and incubated at 50°C for 1 h. DNA was extracted with phenol-chloroform and ethanol precipitation. Libraries were generated using Ad1_noMX and Ad2.3–2.8 barcoded primers from (29) and amplified for 14 cycles. DNA purification was carried out using sparQ PureMag Beads (Quantabio). Primary antibodies against the following proteins were applied: H3K4me1 (1:100, C15410037, Diagenode), H3K27ac (1:100, ab4729, Abcam), H3K27me3 (1:100, C15410069, Diagenode) and H3S28P (1:100, ab5169, Abcam). Two biological replicates were sequenced.

### ATAC-seq

ATAC-seq was performed as described (29) with minor modifications. 100 000 cells were used per experiment. Libraries were generated using Ad1_noMX and Ad2.1–2.2 barcoded primers from (29) and amplified for 10 cycles. DNA was purified using sparQ PureMag Beads (Quantabio) following the manufacturer's protocol for size selection and library preparation. Two biological replicates were sequenced.

### Data processing and analysis of CUT&RUN, CUT&Tag, and ATAC-seq data

Reads were mapped to the hg19/GRCh37 genome using BWA-MEM aligner version 0.7.13 (30). The BAM files were filtered using the encode_task_filter.py script from the ENCODE chip-seq-pipeline2 (https://github.com/ENCODE-DCC/chip-seq-pipeline2/blob/master/src/encode_task_filter.py). Briefly, duplicate reads were marked using Picard version 2.1.1 (https://github.com/broadinstitute/picard) and unmapped, unpaired, low quality (MAPQ < 5), non-primary alignment and duplicate reads were removed using SAMtools version 1.3 (31) with the SAM flag filter ‘-F 1804’. MACS2 was used to call peaks using the parameters ‘-p 0.01 -f BAMPE -g hs’ for the CUT&RUN and CUT&Tag data, and ‘-p 0.01 -g hs –nomodel –shift -75 –extsize 150’ for the ATAC-seq data (32). Bigwig files were generated from the filtered BAM files using MACS2 bdgcmp using the fold enrichment option for the ATAC-seq data (Zhang *et al.* 2008) and deepTools bamCoverage with the options ‘–binSize 1 –normalizeUsing RPGC –effectiveGenomeSize 2864785220 –extendReads’ for the CUT&Tag and CUT&RUN data (33). All bigwig and bed files were filtered using the ENCODE Blacklist (https://github.com/Boyle-Lab/Blacklist/blob/master/lists/hg19-blacklist.v2.bed.gz). Only peaks with *P*-value < 0.00001 were considered for further analyses. EChO 1.0 was performed as described (34). Input fragment bed files were generated from prefiltered paired-end BAM files following the steps provided at https://github.com/FredHutch/EChO using samtools 1.6 (30) and bedtools v2.27.1 (35). Bigwig and bed files were analysed using IGV (36), Galaxy (37) and Cistrome (38). Chromatin states were identified using bedtools intersect intervals of 1 kb spanning the summit of H3K4me1, H3K27ac or H3K27me3 CUT&Tag peaks (35). Differential chromatin accessibility was determined by using bigwigCompare (33) with 1 kb averaging length. Regions with >2-fold increase or decrease in ATAC signal and no called peaks in either the empty vector control cells (for increased chromatin accessibility) or SOX9 OE cells (for decreased chromatin accessibility) were included for further analyses. Motif search was performed using HOMER (39) and the findMotifsGenome.pl command. *De novo* motif search was done for five motifs for each motif length from 6 to 16 bp, with the region for motif finding set at 300 bp. The default HOMER known.motifs file was extended by adding SOX dimer position weight matrices obtained from cisbp (M09387_2.00 and M05761_2.00) (40) with a detection threshold set to 6. HOMERs findMotifsGenome was used with the -find command to find SOX monomer and dimer motifs in given bed files. Motif locations were converted to genomic positions using R. K-means clustering of SOX9 binding modes was performed using ChAsE (41). Heatmaps and average profile plots were generated in ChAsE and Cistrome. Average signals were computed from bigwig files with deeptools multiBigwigSummary (33). GREAT was used to identify genes associated with regulatory regions (42).

### Analysis of scRNA-seq and scATAC-seq data

Raw data scRNA-seq files (ENA accession: PRJNA64623 and PRJNA646233) were processed and aligned to the GRCm38 (mm10) mouse reference genome using Cell Ranger Software (v 6.0.1). Cells with low complexity (<800 expressed genes) and mitochondrial gene expression fractions >15% were excluded using the scater R package (43). Doublets were removed with the doubletCells() function in the Scran R package (44). In addition, clusters of hematopoietic cells were removed. UMAPs and violin plots were generated using scater. Mouse genes were converted to human genes using biomaRt Bioconductor package (45) and correlation with differential gene expression in HUVECs was determined with GSEA (26). After quality control, scRNA-seq data was analysed with the Seurat R package (v3.2.3) as described in (46). Raw scATAC-seq data files (SRA accession: PRJNA646233) were processed with Cell Ranger ATAC Software as described (46). The filtered_peak_bc_matrix.h5, fragments.tsv.gz and singlecell.csv files generated by the cellranger-atac count command were further processed using Signac (v 0.2.5) as described (46). Clusters of hematopoietic cells were removed. The scRNA-seq and scATAC-seq datasets were individually analysed then integrated using Seurat (v3.2.3) (https://github.com/JoLab-Emory/SingleCell/blob/master/scRNA_scATAC_Integration/Integration_Script.txt). UMAPs, violin plots and coverage plots were generated using Seurat. Motifs were identified using HOMER (47) by scanning 300 bp spanning the scATAC peaks.

### Peptide binding assays

MODified™ histone peptide array (13005, Active Motif) was blocked using 3% non-fat milk in TBS and incubated with 2 μg recombinant SOX9 protein (ab131911, Abcam) at 4°C overnight in CHAPS immunoprecipitation buffer (Fivephoton Biochemicals). After washing in TBST, the array was incubated with SOX9 antibody (1:2000, AB5535, Millipore) in 3% non-fat milk in TBS at 4°C overnight, washed in TBST, and incubated with HRP-conjugated secondary antibody in 3% non-fat milk in TBS for 1 h at RT. After washing in TBST, HRP activity was detected with Pierce ECL Western Blotting Substrate (Thermo Fisher Scientific) and imaged using ChemiDoc Imaging System (Bio-Rad) and analysed using Active Motif software.

### Proximity ligation assay (PLA)

E12.5 heart tissue sections and HUVECs were permeabilized with 0.01% Triton-X in PBS. PLA was performed according to the manufacturer's instructions, using Doulink in situ PLA kit (DUO92102 and DUO92106, Sigma-Aldrich). Images were captured using a Nikon Instruments Eclipse Ti confocal laser microscope. Primary antibodies used were: goat anti-SOX9 (1:50, AF3045, R&D Systems), rabbit anti-H3K4me1 (1:50, C15410037, Diagenode), rabbit anti-H3K4me3 (1:50, 9751S, Cell Signaling), rabbit anti-H3K27ac (1:50, ab4729, Abcam), rabbit anti-H3S28P (1:50, ab5169, Abcam), and mouse anti-H3 (1:50, 39763, Active Motif).

## RESULTS

### SOX9 expression is induced in endothelial cells during EndMT *in vivo*

**Figure 1.**
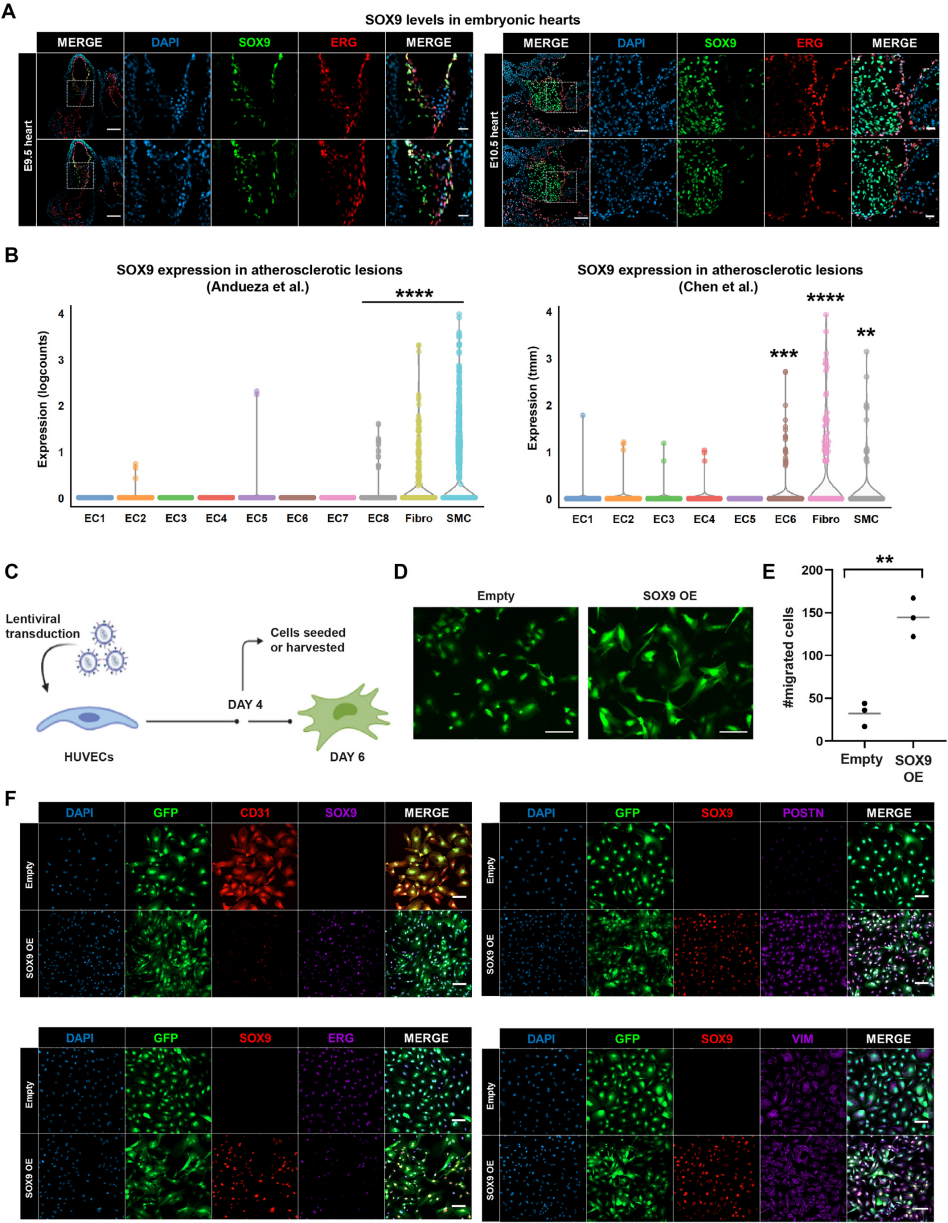
SOX9 is expressed at the onset of EndMT in mouse embryonic hearts and atherosclerotic lesions and ectopic SOX9 expression in human endothelial cells induces a mesenchymal phenotype. (**A**) SOX9 and ERG immunostaining of mouse hearts at E9.5 and 10.5. Scale bar whole hearts, 100 μm. Scale bar areas of interest, 25 μm. (**B**) SOX9 expression in each cluster from Andueza *et al.* (left) and Chen *et al.* (right). Significance was evaluated between clusters EC8, Fibro, or SMC and EC1-EC7 (Andueza *et al.*) and between EC6, Fibro or SMC and EC1-EC5 (Chen *et al.*) by Wilcoxon rank sum tests with *P*-values (*P < 0.05; **P < 0.01; ***P < 0.001; ****P < 0.0001; ns *P* > 0.05). (**C**) Schematic overview of experimental setup. Created with Biorender. (**D**) Fluorescence microscopy of HUVECs 6 days after transduction with SOX9 or empty vector, demonstrating the change in morphology with SOX9 expression. Scale bar, 100 μm. (**E**) Transwell assay indicating number of migrated HUVECs after transduction with SOX9 or empty vector. Significance was evaluated by unpaired, two-tailed *t*-tests with *P*-values (*P < 0.05; **P < 0.01; ***P < 0.001; ****P < 0.0001; ns P > 0.05). Mean is indicated by gray line. (**F**) SOX9, ERG, CD31, POSTN and VIM immunostaining of HUVECs 6 days after transduction with SOX9 or empty vector. Scale bar, 100 μm.

Mouse embryonic heart valve formation initiates when endothelial cells of the heart transition into mesenchymal cells and migrate to populate the cardiac cushions, the precursors of cardiac valves. We previously showed that SOX9 expression is crucial for regulation of gene expression in mouse cardiac cushion mesenchyme at embryonic day (E) 12.5 (17). To determine whether SOX9 is expressed in endothelial cells in the heart undergoing EndMT, we examined SOX9 expression patterns at E9.5 in mouse hearts. At E9.5, the endothelial master TF ERG was expressed in all endothelial cells and downregulated within emerging mesenchymal cells, which expressed SOX9 as they underwent EndMT and populated the cardiac cushions (Figure 1A, left panel). Interestingly, many endothelial cells lining the cushions expressed both SOX9 and ERG, suggesting SOX9 expression is induced in endothelial cells lining the cardiac cushions, prior to overt EndMT, and continues to be expressed through the transition into migrating mesenchymal cells. By E10.5, we detected fewer cells co-expressing SOX9 and ERG, and the SOX9-expressing mesenchymal cells had proliferated, populating the cardiac cushion (Figure 1A, right panel). By E12.5, we observed a further expansion of the mesenchyme consisting of SOX9-expressing cells (Supplementary Figure S1A).

In addition to being crucial for heart valve development, EndMT can be initiated in adults by tissue damage or inflammation and contribute to diseases such as atherosclerosis, pulmonary hypertension, valvular disease, and fibroelastosis (48). To investigate whether SOX9 is expressed upon EndMT in disease, we analysed two available single-cell (sc) RNA-seq datasets consisting of cells from atherosclerotic lesions. Andueza *et al.* (46) performed partial carotid ligation on mice, which exposes the arteries to disturbed blood flow to induce atherosclerosis. Chen *et al.* (49) placed Apolipoprotein-E deficient mice on a high cholesterol, high fat diet (HCHFD) for four months to induce atherosclerosis. We reanalysed the data and identified the same eight endothelial clusters (EC1–EC8) as described in (46), vascular smooth muscle cells (SMC), and fibroblasts (Fibro) (Supplementary Figure S1B and C, left panel), while from the Chen *et al.* data we identified six endothelial clusters (EC1–EC6), SMC and Fibro (Supplementary Figure S1B and C, right panel). In both datasets the endothelial cell clusters consisted of a mixture of cells originating from normal arteries and atherosclerotic lesions, while the mesenchymal cells (Fibro and SMC) mostly originated from arteries exposed to disturbed blood flow from the Andueza *et al.* dataset and a mixture of cells originating from mice on a normal diet or HCHFD from the Chen *et al.* dataset (Supplementary Figure S1D). Interestingly, one large endothelial cell cluster in both datasets almost exclusively consisted of cells from atherosclerotic lesions, EC8 and EC6, from Andueza *et al.* and Chen *et al.*, respectively. Of note, SOX9 transcripts were detected at significant levels in EC8, Fibro and SMC clusters in the Andueza *et al.* data and EC6, Fibro, and SMC clusters in the Chen *et al.* data (Figure 1B). Furthermore, the mesenchymal markers MMP2, TGFBI and VCAN, were also expressed significantly higher in the endothelial clusters containing SOX9-expressing cells compared to the other endothelial clusters (Supplementary Figure S2A).

The above results confirm that SOX9 expression is induced in endothelial cells and plays a role in the early steps of EndMT both during embryonic heart valve development and in atherosclerotic lesions.

### Ectopic SOX9 expression in endothelial cells promotes a mesenchymal phenotype

The functions of SOX9 in EndMT are poorly understood. To determine if ectopic SOX9 expression in endothelial cells would force cells to adopt a mesenchymal phenotype, we used HUVECs, a recognized human EndMT model that lacks endogenous SOX9 expression (50–52). We transduced HUVECs with constructs encoding human SOX9 or an empty vector control, both containing a green fluorescent protein (GFP) reporter gene (Figure 1C and Supplementary Figure S2B). By six days post-transduction the cellular morphology changed from the cobblestone-like shape of endothelial cells to an elongated spindle-shape characteristic of mesenchymal cells (Figure 1D). Notably, the cells could be passaged without reverting to their original morphology, suggesting the mesenchymal morphology induced by SOX9 was permanent and robust. As expected, increased migratory properties (Figure 1E) and downregulated expression of the endothelial surface protein CD31 as well as ERG occured in SOX9-expressing cells (Figure 1F). These cells also had high Periostin levels, while Vimentin levels were slightly higher. This suggests that ectopic expression of SOX9 in endothelial cells is sufficient for and efficient at direct reprogramming to a mesenchymal cell fate.

### SOX9 alters expression of EndMT genes by inducing a switch in chromatin states

**Figure 2.**
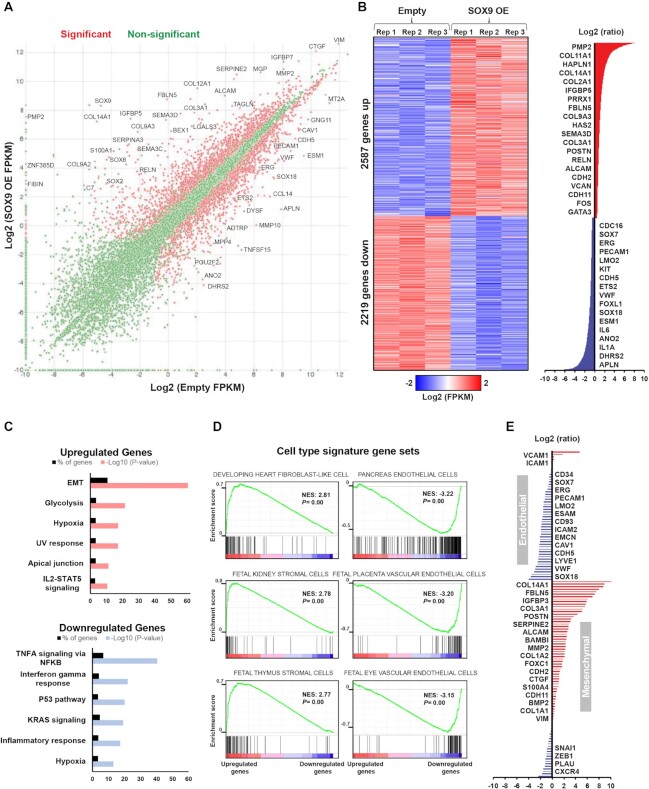
SOX9 alters expression of EndMT genes. (**A**) Scatterplot comparing normalized average log_2_ FPKM values between cells transduced with empty vector or SOX9. Significantly differentially expressed genes are coloured red with select genes highlighted. (**B**) Heatmap of differentially expressed genes between cells transduced with SOX9 or empty vector. Heatmap shows log_2_ FPKM values while log_2_ ratios are displayed on the right with select genes highlighted. (**C**) Gene set enrichment analysis (GSEA) of enriched Hallmark gene sets (based on *P*-values) for upregulated and downregulated genes upon SOX9 expression. (**D**) GSEA cell signatures of genes upregulated (left) and downregulated (right) in HUVECs. (**E**) Log_2_ ratios of SOX9 OE over empty vector for annotated endothelial- or mesenchymal-specific genes with select genes highlighted.

To investigate global transcriptional changes caused by ectopic SOX9 expression in the transition towards mesenchymal cell identity, we employed RNA-seq using HUVECs harvested four days after transduction with SOX9. 2587 genes were upregulated and 2219 genes were downregulated with SOX9 expression (log_2_ ratio > 0.5 or ←0.5, *P*-value < 0.05) (Figure 2A, B and Supplementary Figure S2C). Among the most upregulated genes were components of ECM, including collagens, as well as mesenchymal markers like Periostin (*POSTN*), N-cadherin (*CDH2*) and Cadherin-11 (*CDH11*). Downregulated genes included endothelial markers like von Willebrand factor (*VWF*), VE-cadherin (*CDH5*), and CD31 (*PECAM1*), as well as some key endothelial-associated TFs, such as SOX7, SOX18, ERG and LMO2 (Figure 2B, right panel). Genes upregulated in response to SOX9 were significantly enriched for hallmark gene sets for EMT (Figure 2C) and cell type signature gene sets of mesenchymal cells, with fibroblast-like cell as the top enriched cell type (Figure 2D). Downregulated genes were enriched for inflammatory signaling pathways and cell type signature gene sets of endothelial cells. Although EMT and EndMT are similar processes, gene sets for EndMT currently do not exist in gene ontology (GO) databases. Therefore, we generated a list of endothelial and mesenchymal genes, including genes previously shown to be down- or up-regulated upon EndMT (Supplementary Table S1). SOX9 expression resulted in enhanced expression of mesenchymal genes, with concurrent downregulation of endothelial gene expression (Figure 2E). Of note, we observed a downregulation of some genes encoding TFs known to have a role in EMT, like *SNAI1* and *ZEB1*, indicating that upregulation of mesenchymal markers does not depend on these TFs in this context.

We compared differentially expressed genes in SOX9-expressing HUVECs to differentially expressed genes between fibroblasts and endothelial cells from the scRNA-seq data in atherosclerotic lesions. Genes upregulated by SOX9 expression in HUVECs overlapped with genes upregulated in fibroblasts versus endothelial cells in atherosclerotic lesions, while genes downregulated by SOX9 expression in HUVECs were enriched in endothelial cells versus fibroblasts in atherosclerotic lesions (Supplementary Figure S2D and S2E). This suggests that gene programs activated by SOX9 expression in HUVECs are similar to those that drive EndMT in atherosclerosis.

**Figure 3.**
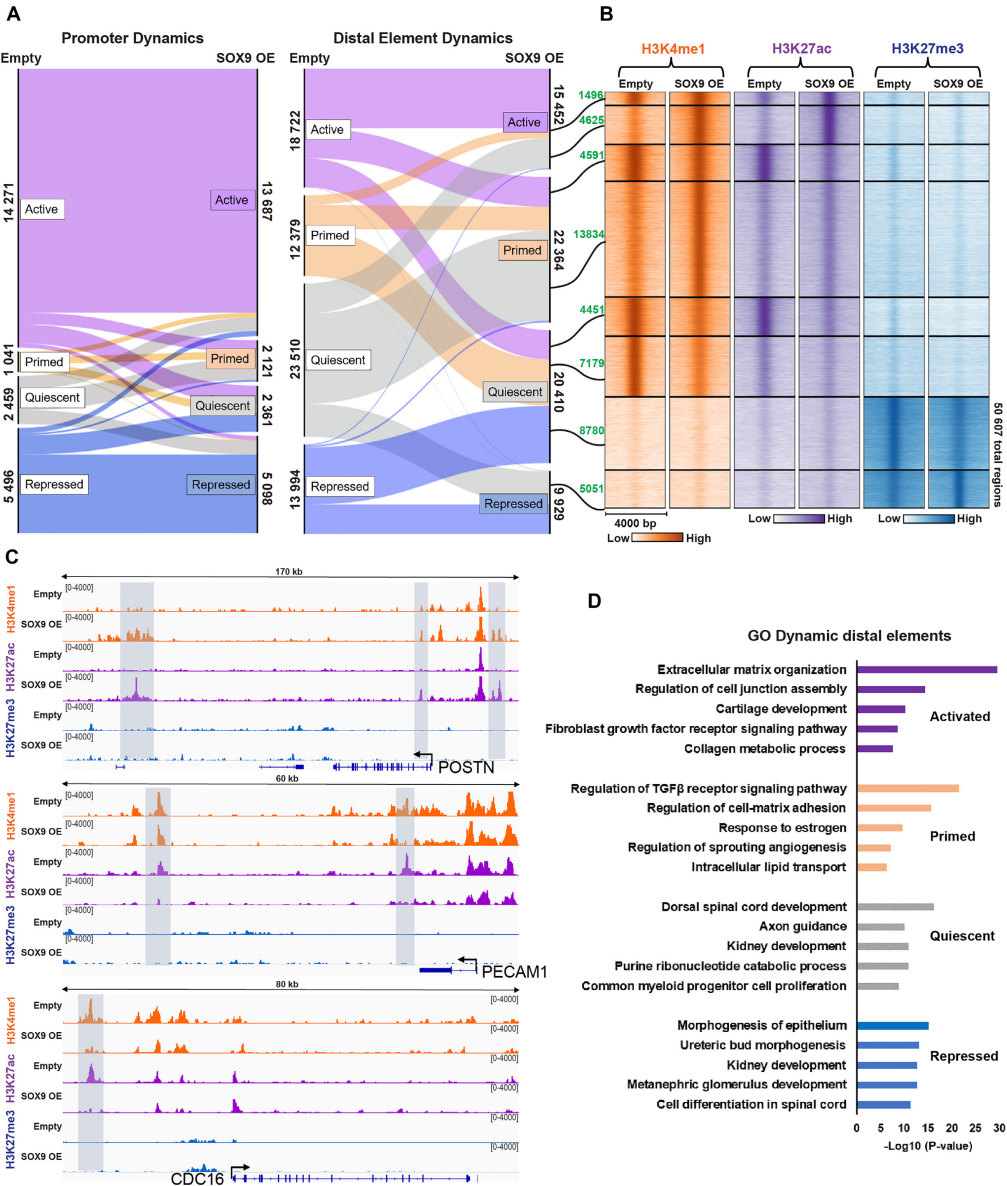
SOX9 expression induces a switch in chromatin states. (**A**) Alluvial plot showing dynamics in chromatin states between cells transduced with SOX9 or empty vector. Alluvia are coloured based on the empty vector state annotations. The number of promoters or distal elements in each alluvium is indicated. (**B**) Heatmap displaying H3K4me1, H3K27ac and H3K27me3 signal within a 4 kb window of dynamic distal elements identified in A). The eight largest alluvia representing dynamic distal elements are shown as separate clusters. The number of genomic regions in each cluster is annotated in green text. (**C**) IGV browser tracks of representative loci displaying a switch in chromatin states: from quiescent to active (POSTN), active to primed (PECAM1), and active to quiescent (CDC16). Dynamic chromatin regions are highlighted with gray boxes. (**D**) Top enriched biological processes for genes associated with dynamic distal elements.

To explore the mechanisms of SOX9 in driving EndMT, we investigated chromatin states and examined the global dynamics of histone modifications after SOX9 expression. Cells were harvested four days after transduction and we used CUT&Tag to profile histone modifications known to be present at regulatory elements: H3K4me1, H3K27ac and H3K27me3. We defined promoters as being <5000 bp upstream or 500 bp downstream from a transcriptional start site (TSS) while we considered elements further away from the TSS as distal elements. We classified chromatin states as active (H3K4me1 + H3K27ac), primed (H3K4me1 only), quiescent (no marks), or repressed (H3K27me3). SOX9 expression caused a major switch in chromatin states of distal elements; in total 50607 regions changed chromatin state (Figure 3A and B). 6298 regions that were primed, quiescent, or repressed in endothelial cells prior to SOX9 expression, were activated with SOX9 expression, whereas 9118 regions that were active in endothelial cells changed state. Promoters did not show the same level of histone modification dynamics (Figure 3A, left panel). The genes associated with activated distal elements were enriched for GO terms for mesenchymal functions, whereas the regions that lost active histone marks were linked with endothelial functions (Figure 3D).

Together, our analysis of the transcriptome and epigenetic landscape show that SOX9 expression is sufficient to induce EndMT by causing a switch in chromatin states. This leads to activation of mesenchymal genes and downregulation of endothelial genes.

### SOX9 direct target genes have known roles in EndMT

To examine the regulatory role of SOX9 in directing EndMT, we used CUT&RUN to profile SOX9 chromatin binding genome-wide in HUVECs four days after transduction. We identified 3473 SOX9 bound regions (Supplementary Figure S3A). Approximately 6% of all SOX9 bound regions were either directly over a TSS or in the 5 kb proximal promoter regions (Supplementary Figure S3B). About 45% of bound regions were located within gene bodies, whereas 49% were distal, indicating that in HUVECs, SOX9 primarily binds distal regulatory elements inside or outside gene bodies.

We used GREAT (42) to determine genes associated with SOX9 bound regions (Supplementary Figure S3C). Genes with both a SOX9 bound region and differential gene expression were considered direct transcriptional targets of SOX9. Of these, 543 genes (63%) were upregulated by SOX9, while 324 genes (37%) were downregulated. Among the upregulated genes were previously characterized SOX9 target genes, such as ECM genes encoding collagens IX and XI (53), as well as other TF genes like SOX5, SOX6, CREB3L2, and NFIA, highlighting the role of SOX9 as a master regulator (17,54,55). Of note, we did not observe SOX9 bound to the known enhancer of the well-characterized SOX9 target gene, COL2A1, although this gene was highly upregulated with SOX9 expression. Other upregulated target genes include the Homeobox TF genes PRRX1 and PKNOX2, both of which have roles in mesenchyme development (56,57), while PRRX1 also promotes EMT in cancers (58,59). Genes possibly repressed by SOX9 include endothelial markers, such as *VWF* and *PECAM1*, as well as genes encoding TFs important for endothelial cell functions, including ERG (60) and ETS2 (61).

TF binding to chromatin can be categorized by enhanced chromatin occupancy (EChO), a computational strategy shown to distinguish direct TF binding to DNA from nucleosomal binding by analysing average fragment size of called peaks in CUT&RUN data as this reflects sites of minimal DNA protection by proteins (34). Using EChO within our SOX9 data, we observed a distribution of fragment sizes mostly ranging between 50 and 150 bp, indicating binding by SOX9 to both nucleosomes and open DNA (Supplementary Figure S3D). Searching for enriched DNA binding motifs in the SOX9 peaks, we identified SOX motifs as the top three enriched (Supplementary Figure S3E), including two dimer (one defined and one more degenerate) and one monomer SOX motif, which are similar to previously identified motifs for SOX9 (5,17,53,62).

In summary, we identified direct SOX9 target genes, most of which were upregulated by SOX9 and many of which have known roles in mesenchymal cells.

### SOX9 opens chromatin at a subset of binding sites in closed chromatin

**Figure 4.**
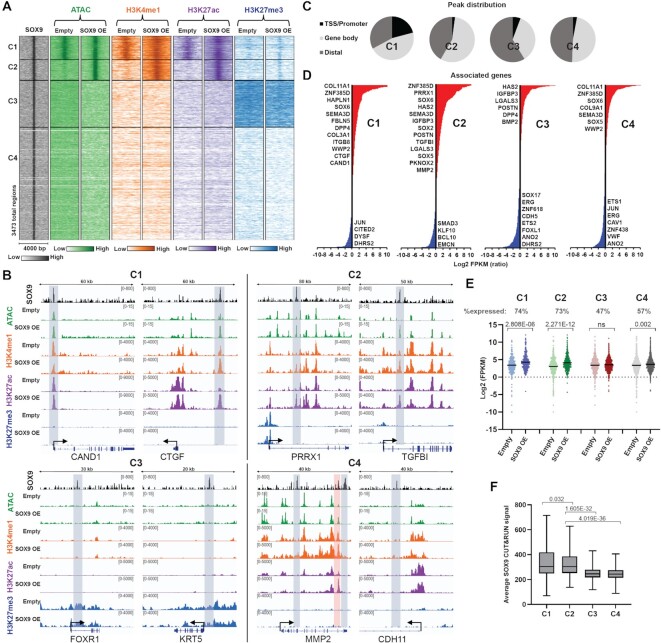
SOX9 opens chromatin and increases enrichment of active histone modifications at a subset of binding sites. (**A**) Heatmap displaying SOX9, ATAC, H3K4me1, H3K27ac and H3K27me3 signal within a 4 kb window around the summit of SOX9 bound regions. K-means clustering revealed four modes of SOX9 binding (C1–C4). (**B**) Representative loci from each cluster. Regions with a SOX9 peak are highlighted with gray boxes, and the C2 region in the MMP2 locus is highlighted with a red box. (**C**) Distribution of SOX9 bound regions between TSS/promoter regions (5000 bp upstream to 500 bp downstream of TSS), within gene bodies, or distal. (**D**) Log_2_ FPKM ratios for differentially expressed genes with an associated SOX9 bound region in C1–C4. Select genes are highlighted. (**E**) Scatterplots of log_2_ FPKM values of expressed genes (% shown) with an associated SOX9 bound region in C1–C4. Significance was evaluated as in Figure 1E. Mean is indicated with black line. (**F**) Average SOX9 CUT&RUN signal at the summits of SOX9 bound regions in C1–C4. Significance was evaluated as in Figure 1E. Mean is indicated with black line.

Since SOX9 primarily bound to distal regulatory elements (including potential enhancers), we examined which chromatin states allow occupancy by SOX9 and if SOX9 can alter the chromatin landscape. To determine the impact of SOX9 on chromatin accessibility, we performed ATAC-seq in HUVECs four days after transduction and correlated the data with H3K4me1, H3K27ac, and H3K27me3, described above. We classified SOX9 bound regions into four distinct clusters, termed C1–C4 (Figure 4A and Supplementary Figure S3F). The first cluster, C1 (12%), contains regions with high chromatin accessibility regardless of SOX9 presence, as in the promoter of the gene encoding CAND1 and the distal region upstream of the gene encoding the ECM-associated protein CTGF (Figure 4B). We also observed high levels of H3K4me1 and H3K27ac in these regions, indicative of active regulatory regions, which increased slightly with SOX9 occupancy. C2 regions (10%) had closed chromatin and no enrichment of the assayed modifications, but with SOX9 bound they gained high chromatin accessibility and enrichment of H3K4me1 and H3K27ac. These newly opened chromatin regions suggest pioneer TF activity by SOX9. As shown at the PRRX1 and TGFBI loci, these regions gained chromatin accessibility and active histone modifications with SOX9 expression. C3 (23%) consisted of regions in closed chromatin and no active histone modifications but high enrichment of the repressive H3K27me3, as shown in the FOXR1 and KRT5 loci. Neither chromatin accessibility, active histone modifications, nor H3K27me3 levels were affected by SOX9 binding. The final C4 cluster, composed of over 50% of SOX9 bound regions, was closed with low levels of histone modifications and did not show changes with SOX9 binding. The MMP2 locus is an interesting example as it had two C4 regions in addition to a C2 region and this gene was highly upregulated with SOX9 expression.

Although most SOX9 binding events occurred in distal regions, C1 had a larger proportion of sites in promoters and/or near the TSS of genes compared to the other clusters (Figure 4C and Supplementary Figure S4A). Chromatin changes induced by SOX9 in C1 and C2 regions lead to an overall upregulation of the associated genes (Figure 4D, E, and Supplementary Figure S4B). Expression of genes associated with C3 regions did not significantly change. Of note, many genes associated with C4 regions also had C1 or C2 type SOX9 binding (Supplementary Figure S4C). After removing all expressed genes that overlap with C1 and/or C2 genes from C4 genes (18%), there was no significant change in expression of the remaining C4 genes (Supplementary Figure S4D). While over 70% of genes with C1 or C2 type SOX9 binding were expressed, only ∼50% of C3 or C4 genes were expressed in either condition (Figure 4E). As pioneer TFs activate genes in silent chromatin, we asked whether C2 regions, where SOX9 may open chromatin, are associated with more genes that were silent prior to SOX9 binding compared to the C1 group. This was indeed the case, as 17% of the upregulated genes associated with C2 regions were silent prior to SOX9 binding, while only 7% of the upregulated C1 genes were silent before (Supplementary Figure S4E).

Genes associated with C1 and C2 regions were enriched for processes connected with mesenchymal cell identity, with C2 regions most enriched for developmental processes and C1 enriched for cellular organization (Supplementary Figure S4F). The latter may reflect that C1 genes were already expressed prior to SOX9 binding, leading to further upregulation. In contrast, genes associated with C2 regions were more specifically linked to a transition to a mesenchymal fate. Notably, we did not identify significant biological processes associated with the regions in C4, although this was the largest of the four clusters, suggesting that this cluster represents non-specific binding or scanning by SOX9 (63). We observed lower average SOX9 signal in C3 and C4 regions compared to C1 and C2, suggesting the former are lower-affinity binding events (Figure 4F), although SOX9 must be pausing at these sites to permit significant detection.

Taken together, our profiling of the chromatin landscape shows that SOX9 binds to active and open chromatin regions as well as to closed and silent chromatin. At a subset of these silent regions, SOX9 increases chromatin accessibility, suggesting a role as a pioneer TF. The regulatory elements activated by SOX9 drive expression of mesenchymal genes and lead to a transition in cell fate.

### SOX dimer motifs are required for SOX9-induced chromatin opening

**Figure 5.**
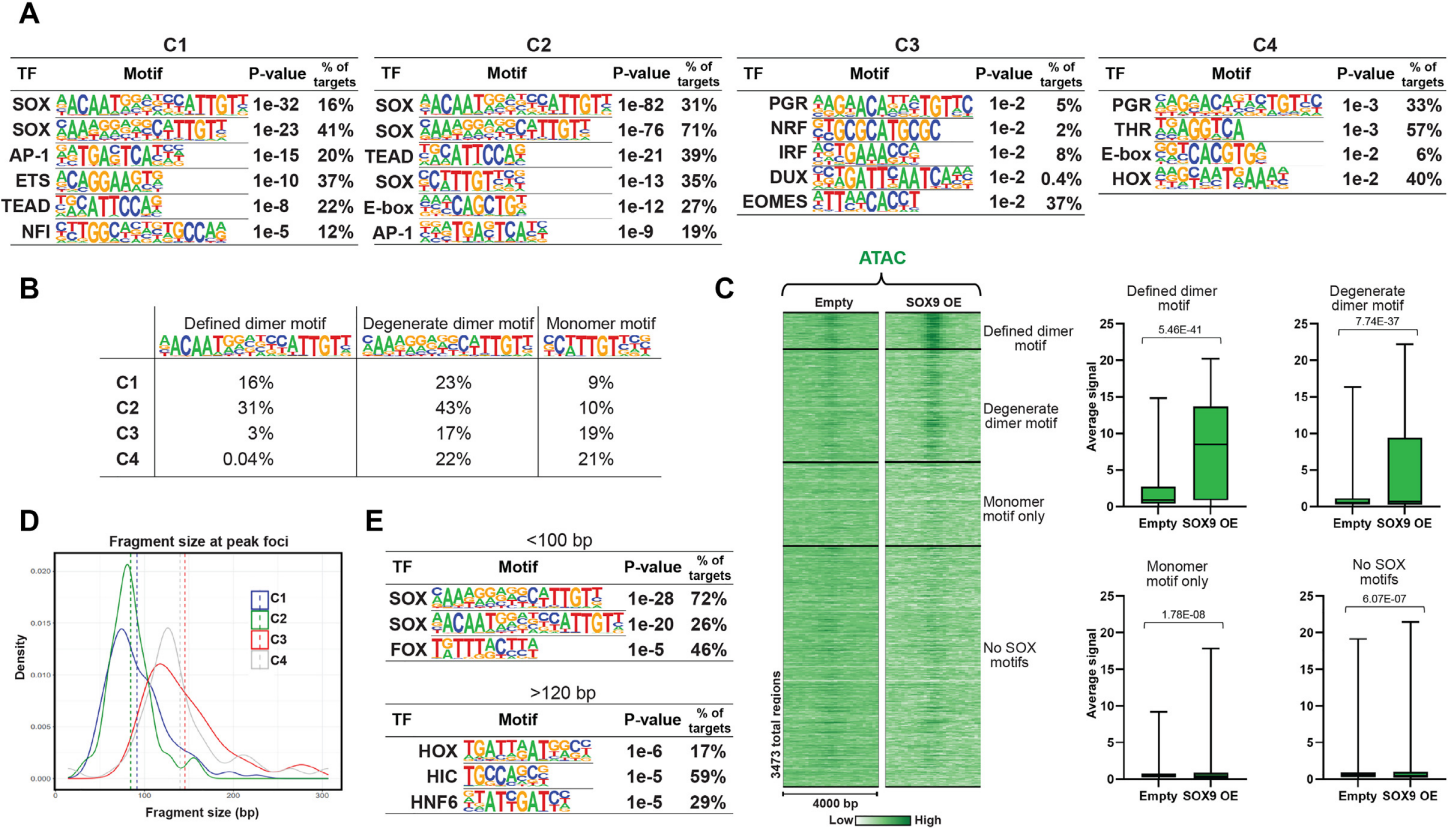
Chromatin opening by SOX9 is motif encoded. (**A**) Top TF motifs enriched (or the only enriched in C3 and C4) in SOX9 bound regions. (**B**) Percentage of SOX motifs in SOX9 bound regions. ‘Degenerate dimer motif’ regions only count occurrences not overlapping with ‘Defined dimer motif’ regions, while ‘Monomer motif’ regions only count occurrences not overlapping with any of the dimer motif regions. (**C**) Left panel: Heatmap displaying ATAC signal within a 4 kb window around the summit of SOX9 bound regions. Regions were clustered according to the occurrence of SOX motifs. Right panel: Average ATAC signal at the summits of indicated SOX9 bound regions. Significance was evaluated as in Figure 1E. Mean is indicated with black line. (**D**) Density plot displaying distributions of average fragment size at detected foci for SOX9 CUT&RUN. Vertical dashed lines represent average fragment size. (**E**) Top TF motifs enriched in foci <100 bp and >120 bp.

As SOX9 binding increased chromatin accessibility at only a subset of its bound regions in closed chromatin, we questioned if DNA sequence might intrinsically function to dictate how SOX9 binding affects chromatin accessibility. Motif analysis revealed that SOX motifs were significantly enriched in C1 and C2 regions, with the highest number in C2 regions (Figure 5A). The defined and the degenerate dimer motifs identified in Supplementary Figure S3E were the top two enriched motifs, with a larger percentage of C2 regions containing these motifs. C2 regions also had a SOX monomer motif enriched as the fourth most significant. C3 and C4 regions did not show evidence of SOX motif enrichment and had fewer motifs significantly enriched overall. This suggests SOX9 binding to closed chromatin, as in C2-C4, is not dictated by SOX motifs, but the ability of SOX9 to open chromatin, as in C2, is dependent on the presence of a SOX motif.

We sorted SOX9 bound regions containing SOX motifs into clusters with the defined dimer motif, the degenerate dimer motif (excluding the ones overlapping with the defined dimer motif) and the monomer motif only (excluding regions overlapping with either of the dimer motifs) (Figure 5B) and examined the ATAC-seq signal (Figure 5C). Strikingly, chromatin opening predominantly occurred in regions containing the defined dimer motif and to a slightly lesser degree the degenerate dimer motif.

EChO analysis for clusters C1–C4 revealed a smaller average fragment size (<100 bp) for the regions in C1 and C2, reflective of direct DNA binding, and a larger average fragment size (∼150 bp) for C3 and C4 regions, suggesting binding by SOX9 to nucleosomal DNA (Figure 5D). This indicates that SOX9 binds directly to DNA in open chromatin regions, including the regions that were opened with SOX9 binding (C2), suggesting that we capture SOX9 binding following chromatin opening in C2. We sorted all the detected foci from our EChO analysis into <100 bp fragments and >120 bp fragments and searched for enriched motifs, which found the SOX dimer motifs enriched in <100 bp foci but not in the > 120 bp foci (Figure 5E).

Our results strongly suggests that SOX9 must dimerize to open chromatin in agreement with the previous study showing that disturbing SOX9 dimerization abolish its ability to remodel chromatin in vitro (18). Furthermore, the presence of monomer motifs in regions where SOX9 is binding and possibly scanning but not opening closed chromatin may explain why SOX9 is pausing at these sites.

### SOX9 binding is dynamic but induces stable changes in the chromatin landscape

**Figure 6.**
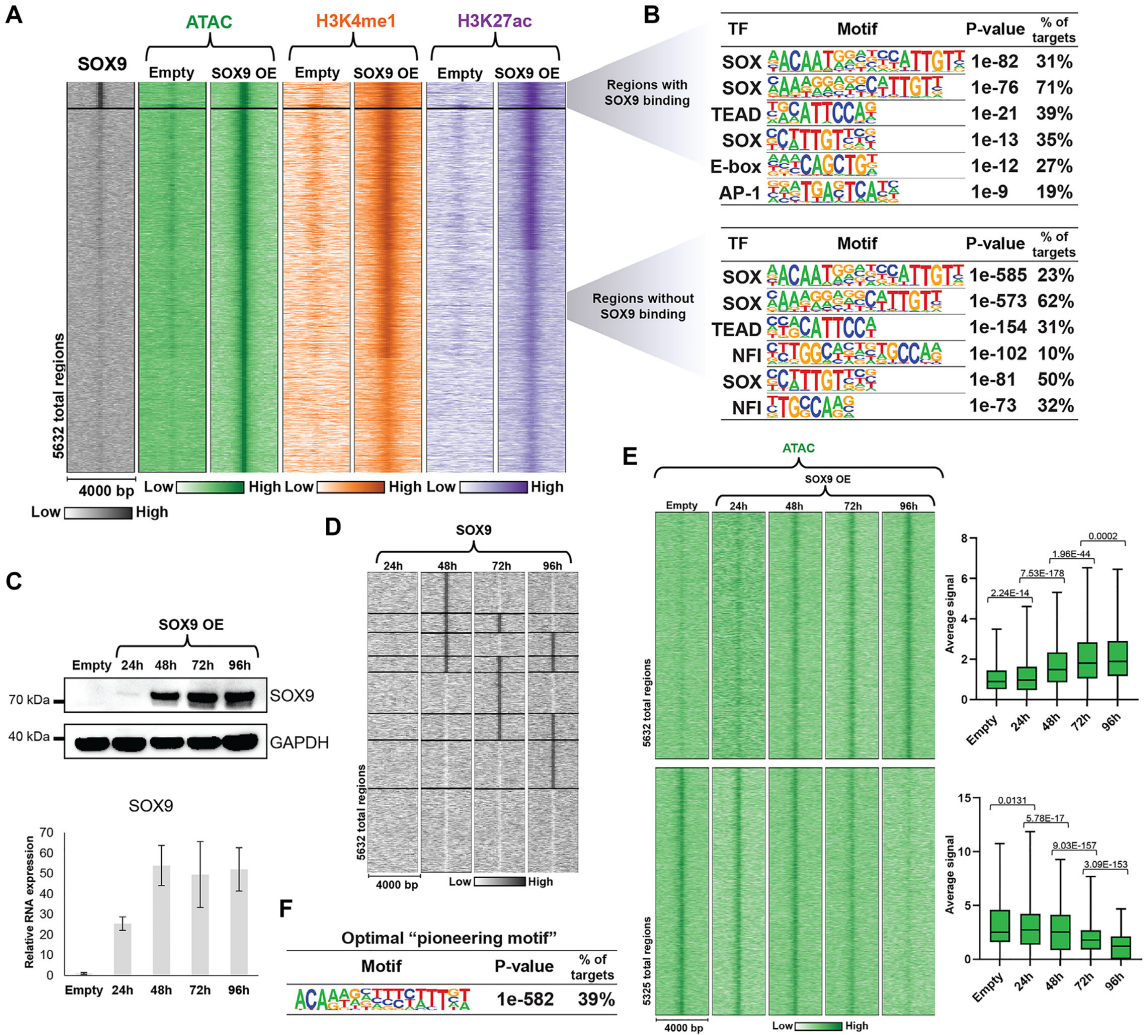
Global changes in chromatin accessibility are caused by dynamic SOX9 binding. (**A**) Heatmap displaying SOX9, ATAC, H3K4me1, and H3K27ac signal within a 4 kb window around the summit of ATAC peaks in regions with increased chromatin accessibility. The top cluster displays regions with SOX9 binding (C2 from Figure 4A). (**B**) Top TF motifs enriched in regions with increased chromatin accessibility with SOX9 binding (top) or without detected SOX9 binding (bottom). (**C**) Expression of SOX9 confirmed by western blotting and RT-qPCR at the indicated timepoints after transduction. (**D**) Heatmap displaying SOX9 signal within a 4 kb window around the summit of ATAC peaks from Figure 6A at the indicated timepoints after transduction. (**E**) Left panel: Heatmap displaying ATAC signal within a 4 kb window around the summit of ATAC peaks in regions with increased chromatin accessibility (top) and decreased chromatin accessibility (bottom) at the indicated timepoints after transduction. Right panel: Average ATAC signal at the summits of ATAC peaks in regions with increased (top) and decreased (bottom) chromatin accessibility at the indicated timepoints after transduction. Significance was evaluated as in Figure 1E. Mean is indicated with black line. (**F**) Top enriched TF de novo motif in regions with increased chromatin accessibility.

To investigate the global impact on chromatin accessibility regardless of SOX9 binding, we examined all regions that gained or lost chromatin accessibility with SOX9 expression (Figure 6A and Supplementary Figure S5A). Regions with altered chromatin accessibility were exclusively distal and enriched GO terms for the associated genes showed a clear link to EndMT processes, highlighting the role of activated distal regions in defining cell phenotype (Supplementary Figure S5B and S5C). Four days after transduction, we only detected SOX9 binding at 6% of the total regions with increased chromatin accessibility, suggesting that either other TFs are responsible for chromatin opening downstream of SOX9 or SOX9 binding at these regions is transient, allowing SOX9 to bind, open and activate chromatin and then leave. Interestingly, after subtraction of detected SOX9 bound regions, SOX dimer motifs were nonetheless the most significantly enriched in the newly opened chromatin (Figure 6B). Of note, other SOXE factors, SOX8 and SOX10, which can bind the SOX9 dimer motif, were not expressed in HUVECs in either condition (not shown). Regions with decreased chromatin accessibility were enriched for motifs for AP-1 family of TFs, such as JUN and FOS, as well as motifs for the endothelial TFs ETS and ERG (Supplementary Figure S5D). Most members of these TF families were highly expressed in HUVECs and downregulated upon SOX9 expression (Supplementary Figure S5E), suggesting that AP-1 and ETS family TFs maintain active enhancers in HUVECs, and downregulation of these factors leads to loss of chromatin accessibility.

Due to the enrichment of SOX dimer motifs in newly opened chromatin regions where we did not observe SOX9 binding four days post-transduction, we asked if SOX9 was bound in these regions at earlier timepoints. SOX9 mRNA and protein were detectable after 24 h (Figure 6C) and by 48 h we detected SOX9 binding in many newly opened regions where we did not detect SOX9 bound at 96 h (Figure 6D). Many opened chromatin regions were bound by SOX9 at 48h only, but we observed multiple patterns of SOX9 binding that were strikingly transient and dynamic. Remarkably, SOX9 binding at the tested timepoints accounted for 67% of the opened regions where we did not detect SOX9 binding in our previous CUT&RUN (96 h post-transduction). These 3548 regions, in addition to the 336 C2 regions, are sites where SOX9 may act as a pioneer TF. Motif analysis of the remaining regions still showed SOX dimer motifs as the top enriched motifs, emphasizing transient chromatin binding by SOX9 (Supplementary Figure S5F).

To determine the timing of chromatin opening and closing, we performed ATAC-seq on cells harvested at 24, 48, 72 and 96 h post-transduction with SOX9 (Figure 6E). Induction of chromatin opening predominantly occurred at 48 h, coinciding with SOX9 chromatin binding. Closing of chromatin, on the other hand, mainly occurred at 96h post-transduction. As we did not detect SOX9 bound to these regions, closing of chromatin is likely a downstream effect of SOX9 action, such as through the downregulation of AP-1 and ETS family TFs.

Since newly opened chromatin regions were potential pioneering sites of SOX9, we performed a de novo motif search to retrieve an optimal ‘pioneering motif’ for SOX9 (Figure 6F). The resulting motif was similar to the defined dimer motif, but slightly more degenerate. Interestingly, one of the half-sites in this dimer motif was lacking the ‘G’ nucleotide from the canonical Ca/tTTGT motif, which has also been observed at sites of nucleosomal binding by the pioneer and pluripotency factor SOX2 (64).

Taken together, we observed transient and dynamic chromatin binding by SOX9. The chromatin changes induced by SOX9 were, however, stable and driving activation of mesenchymal genes and cell fate transition.

### H2A.Z enrichment marks sites of SOX9 pioneering, while H3S28P inhibit SOX9 chromatin binding

**Figure 7.**
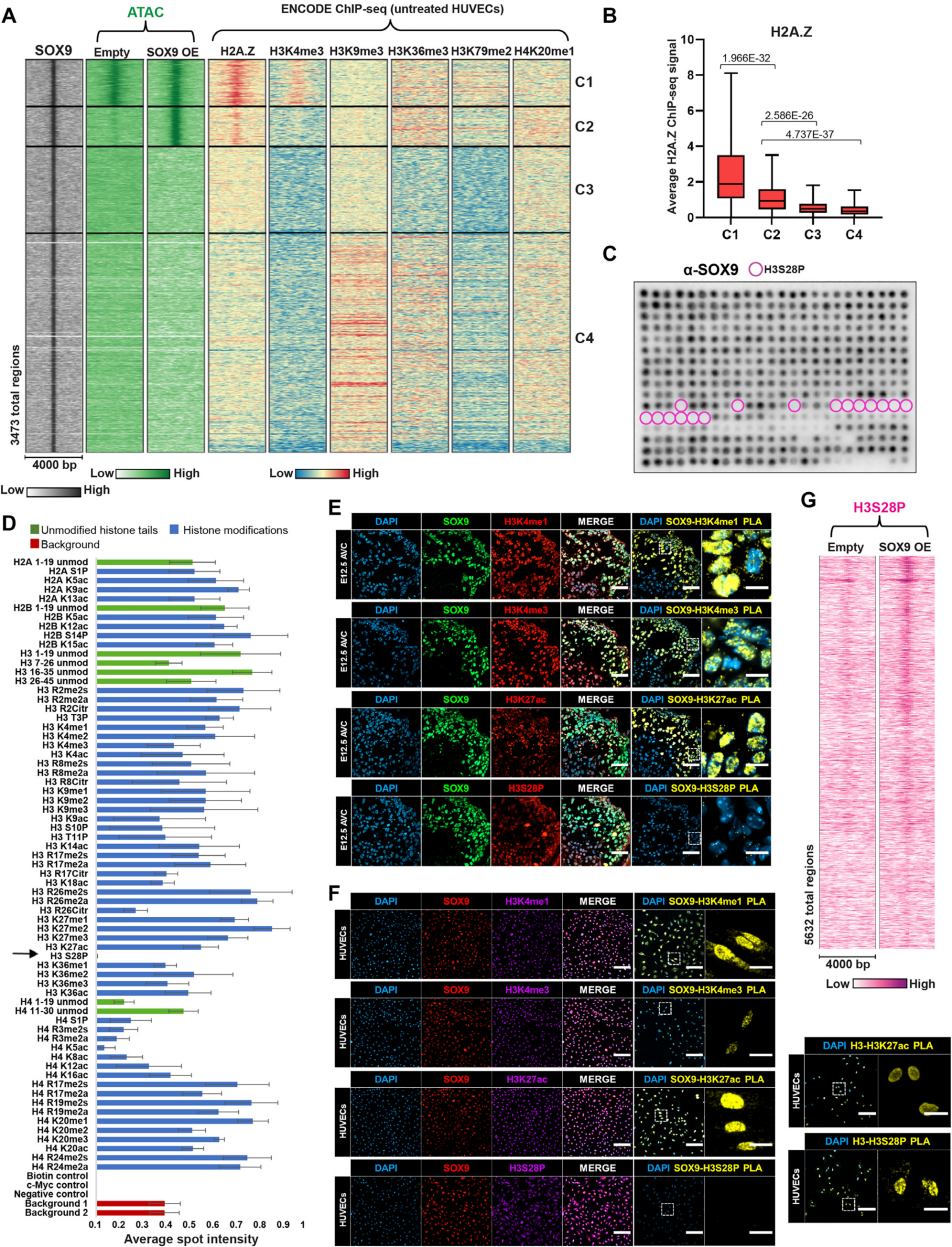
Enrichment of H2A.Z and H3S28P determines SOX9 binding and chromatin opening. (**A**) Heatmap displaying SOX9, ATAC, H2A.Z (ENCODE), H3K4me3 (ENCODE), H3K9me3 (ENCODE), H3K36me3 (ENCODE), H3K79me2 (ENCODE) and H4K20me1 (ENCODE) signal within a 4 kb window around the summit of SOX9 bound regions. (**B**) Average H2A.Z ChIP-seq signal (ENCODE) at the summits of SOX9 bound regions in C1–C4. Significance was evaluated as in Figure 1E. Mean is indicated with black line. (**C**) Histone peptide array containing 384 different histone tail modification combinations incubated with recombinant SOX9 protein and detected with anti-SOX9 primary antibody. Spots containing H3S28P (alone or in combination with other histone modifications) are highlighted with pink circles. (**D**) Relative average spot intensity (from 4 arrays) for all single modified histone peptides (blue), unmodified peptides (green), and background array spots (red). H3S28P is indicated with arrow. (**E**) SOX9, H3K4me1, H3K4me3, H3K27ac and H3S28P immunostaining of mouse atrioventricular canals at E12.5 and PLA signal (yellow) on serial sections showing interactions occurring between SOX9 and the indicated histone modifications. Scale bar, 50 μm. Scale bar areas of interest, 10 μm. (**F**) SOX9, H3K4me1, H3K4me3, H3K27ac and H3S28P immunostaining of SOX9-expressing HUVECs and PLA signal (yellow) showing interactions occurring between SOX9 and the indicated histone modifications. Right panel shows PLAs between H3 and indicated histone modifications as a positive control. Scale bar, 100 μm. Scale bar areas of interest, 25 μm. (**G**) Heatmap displaying H3S28P signal within a 4 kb window around the summit of ATAC peaks in regions with increased chromatin accessibility.

We next asked whether unique chromatin features were enriched in C2, such as histone variants or histone modifications, compared to other SOX9 bound regions in closed chromatin. We explored available datasets from HUVECs deposited by the Encyclopedia of DNA Elements (ENCODE) project (Supplementary Table S2) and discovered C2 regions were enriched for the histone variant H2A.Z (Figure 7A, B, and Supplementary Figure S6A). Although we observed a lower enrichment of H2A.Z in C2 regions compared to the active C1 regions, the presence of H2A.Z in otherwise silent chromatin may be a determinant for SOX9 pioneering. As an example (Supplementary Figure S6B), we detected three SOX9 bound regions upstream of the LGALS3 locus in closed chromatin, but only the region containing H2A.Z became opened with SOX9 binding. Notably, we observed H2A.Z enriched in most sites with increased chromatin accessibility (Supplementary Figure S6C). Furthermore, when we examined all H2A.Z peaks in closed chromatin in HUVECs, we observed increased chromatin opening with SOX9 expression in the regions that also contained a SOX dimer motif (Supplementary Figure S6D). Although we did not detect SOX9 bound at all sites with H2A.Z enrichment and SOX motifs, these are prerequisites for silent chromatin regions to become opened and activated by SOX9. Of note, we observed a higher enrichment of the heterochromatin mark H3K9me3 in C4 regions, suggesting that SOX9 is able to bind, but not open, heterochromatin regions.

We next mapped SOX9 binding to histone tails using a peptide array containing 384 unique histone modification combinations (Figure 7C and Supplementary Figure S6E). Strikingly, SOX9 bound most histone tail modifications as well as the unmodified histone tails (Figure 7C and D). However, H3S28P completely abolished SOX9 binding, both when the modification was present alone and in combination with other histone modifications (Figure 7C, D, and Supplementary Table S3). To examine whether SOX9 association with chromatin is affected by H3S28P in vivo we used proximity ligation assay (PLA). We observed physical association of SOX9 with histone modifications H3K4me1, H3K4me3 and H3K27ac in E12.5 mouse cardiac cushions and in SOX9-expressing HUVECs (Figure 7E and F). However, SOX9 did not associate with chromatin marked with H3S28P in vivo, confirming that this modification may disrupt SOX9 chromatin binding.

By H3S28P CUT&Tag, we detected increased enrichment of H3S28P in sites with chromatin opening upon SOX9 expression (Figure 7G and Supplementary Figure S6F). This suggests that H3S28P, which is associated with open chromatin and activation of transcription (65), may evict SOX9 from nucleosomes following chromatin opening and activation. Thus, while the presence of SOX dimer motifs and H2A.Z in closed chromatin regions is a prerequisite for SOX9 to open chromatin, SOX9 is not dependent on enrichment of specific histone tail modifications to bind chromatin. However, H3S28P may weaken SOX9 binding and evict SOX9 from nucleosomes, contributing to the dynamic SOX9 chromatin binding.

### Possible role of SOX9 in chromatin opening in cells undergoing EndMT in atherosclerosis

**Figure 8.**
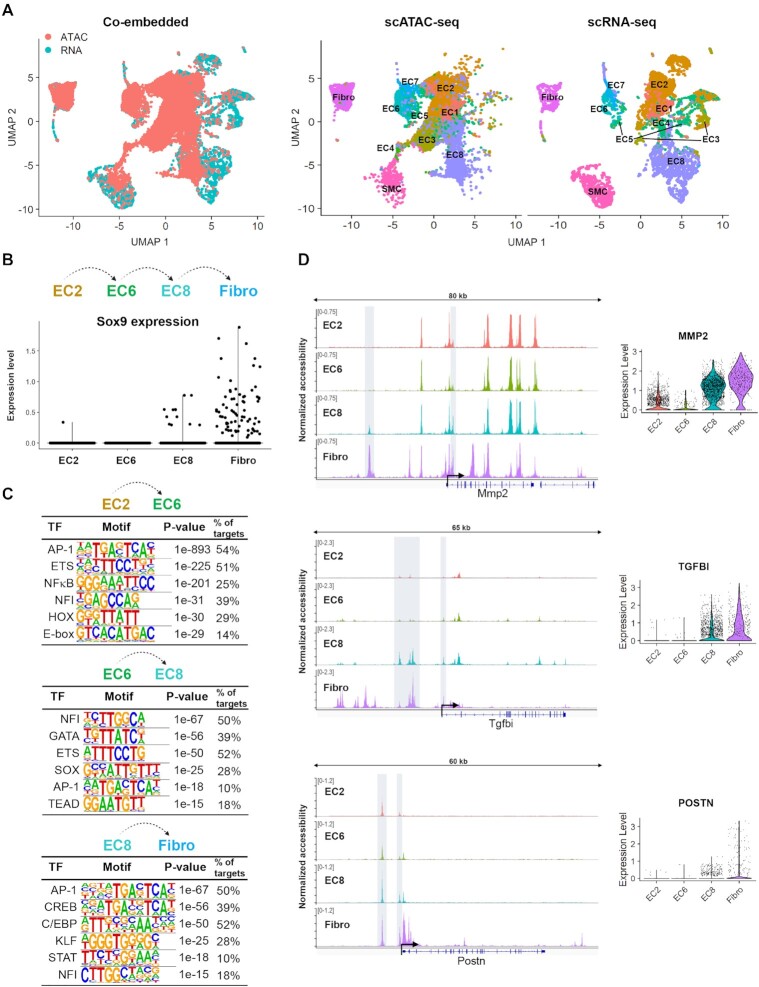
scATAC-seq study suggests SOX9 opens chromatin in cells undergoing EndMT in atherosclerosis. (**A**) UMAP representations of co-embedded and separate scRNA-seq and scATAC-seq mouse atherosclerosis datasets from Andueza *et al.* (**B**) Pseudotime progression of EndMT based on trajectory from Andueza *et al.* and Sox9 expression in each cluster. (**C**) Top enriched motifs in regions that gained chromatin accessibility between EC2 to EC6, EC6 to EC8, and EC8 to Fibro. (**D**) Representative loci with increased chromatin accessibility from EC6 to EC8 (highlighted with gray boxes). Right panel shows the expression pattern of the given genes.

In addition to the scRNA-seq mentioned above, Andueza *et al.* also performed scATAC-seq on atherosclerotic lesions after partial carotid ligation (46). We co-embedded their scRNA-seq and scATAC-seq datasets and analysed differentially accessible regions in the clusters (Figure 8A). Andueza *et al.* generated a pseudotime trajectory which indicated EC8 consisted of cells undergoing EndMT, expressing both endothelial and mesenchymal marker genes in response to chronic disturbed flow, while EC6 represented an earlier intermediate endothelial phenotype, with the Fibro and SMC clusters as the terminally differentiated mesenchymal phenotypes (46). We examined gene expression and chromatin accessibility profiles of EC2 (normal endothelium), EC6, EC8 and Fibro cells, representing progressing stages of the EndMT process (Figure 8B). We identified differentially accessible regions between the four clusters in the scATAC-seq data and searched for motifs in regions that became accessible through EndMT progression. Interestingly, SOX motifs were significantly enriched in regions that became newly accessible in EC8 only (Figure 8C). The SOX dimer motif was not enriched as one of the top motifs but listed further down on the list (*P*-value 1e–4). Some of these opened regions containing SOX motifs included the promoters and putative enhancers upstream of Mmp2, Tgfbi and Postn (Figure 8D), which were direct target genes of SOX9 in HUVECs. Furthermore, genes associated with regions of increased chromatin accessibility in EC8 that contained SOX motifs, were linked to mesenchymal transition (Supplementary Figure S7A). Of note, none of the other SOX factors showed the same expression pattern as SOX9, being unique to EC8 and Fibro (Supplementary Figure S7B), suggesting SOX9 is involved in chromatin opening in endothelial cells in lesions to promote a mesenchymal cell phenotype during atherosclerosis.

## DISCUSSION

Cell reprogramming in embryonic development is crucial for cell fate determination and often involves combinations of TFs. The same pathways governing development of the early embryo are often reactivated in disease. Previous work in mouse has shown that SOX9 is essential for the EndMT process that produces functioning heart valves, as well as in skin wound healing, and acts as a master TF (11,16,17,66). In this study, our analyses provide evidence that SOX9 expression in human endothelial cells can drive EndMT by altering the chromatin landscape. By using genome-wide techniques, we show that SOX9 can induce opening of chromatin and deposition of active histone modifications at previously silent binding sites. The activation of these chromatin regions drives expression of mesenchymal genes, highlighting the ability of one single TF to induce major chromatin restructuring and cell fate decisions. Moreover, by analysing available scRNA-seq datasets, we propose that SOX9 expression in endothelial cells is driving EndMT in atherosclerotic lesions supporting a role of SOX9 in pathological processes.

In HUVECs, SOX9 induced expression of a plethora of TFs associated with mesenchyme, including *PRRX1, PKNOX2*, *FOXC1* and other SOX TFs, such as *SOX5* and *SOX6*. Interestingly, some TFs with known functions in EndMT/EMT, such as *SNAI1* and *ZEB1*, were downregulated in our dataset. Another factor promoting EMT, *SNAI2*, was not expressed in our dataset. Interestingly, both SNAI1 and SNAI2 are required for EndMT in cardiac cushion morphogenesis in mice (67). Our data may suggest that these factors function upstream of SOX9 or parallel to SOX9 and that SOX9 can initiate EndMT independently of these TFs.

Mapping of SOX9 binding and the chromatin landscape identified different modes of SOX9 binding. Surprisingly, very few SOX9 bound regions localized to open chromatin regions in HUVECs and were rarely found in promoter regions of genes. Previous studies of genome-wide chromatin binding by SOX9 found that 20–30% of SOX9 bound regions occurred within TSS/proximal promoter sites in developing mouse heart valves, limbs, and chondrocytes (17,62). In mouse chondrocytes, a SOX motif was enriched in distal regions while promoter regions were enriched for motifs suggesting binding of SOX9 through the basal transcriptional machinery (62). Of note, we observed significantly lower enrichment of SOX motifs in promoter regions of genes compared to distal chromatin regions in our data (Supplementary Figure S7C).

Pioneer TFs have the unique ability to engage their target sites in closed chromatin, render them accessible and allow other factors to bind (68). Our results suggests that SOX9 harbours this ability but, as observed with other pioneer TFs (47,69,70), not all SOX9 binding events result in opening of chromatin. The SOX dimer motif seems to work as an intrinsic signal to guide SOX9 pioneer activity, as pioneering events predominantly occurred at regions with SOX dimer motifs. The pluripotency factors OCT4, SOX2, and KLF4, when acting as pioneer TFs, bind nucleosomes *in vitro* by targeting partial and degenerate DNA motifs displayed on the nucleosome surface (64). According to our data, when SOX9 is acting as a pioneer TF, the optimal pioneering motif is a slightly degenerate dimer motif, supporting a study showing that SOX9 chromatin remodeling of nucleosomes assembled *in vitro* was abolished when SOX9 dimerization was disrupted (18). Interestingly, the same study also showed that SOX9 chromatin binding was unaffected by disrupted dimerization, which is in line with our observation that the monomer motif was more enriched in regions where SOX9 bound but did not open chromatin.

Our time-course revealed that SOX9 chromatin binding is highly dynamic. However, chromatin remained open after SOX9 was no longer bound and cell morphology changes persist. Dynamic chromatin binding has been observed for other pioneer TFs (47) and pioneering may be viewed as a one-time event, meaning that SOX9 does not need to remain bound at sites once opened. Possibly the recruitment of chromatin modifiers and other TFs act downstream of SOX9 to maintain the open chromatin and ensure stable specification of cell identity.

Pioneering by SOX9 predominantly occurred in distal regions with enrichment of the histone variant H2A.Z. H2A.Z has been shown to destabilize nucleosomes and is often found at active promoters and enhancers (71), which we observed in our C1 cluster. The role of H2A.Z in closed chromatin is less clear, but H2A.Z occupancy seems to correlate with pioneer TF binding to nucleosome-occupied regions and has been suggested to act as a scaffold for binding of pioneer TFs and chromatin remodelers (72,73). Whether SOX9 requires destabilized nucleosomes for chromatin opening and activation of target enhancers remains undetermined, but the requirement for H2A.Z enrichment and dimer motif presence is evident from our study. We could not identify specific histone tail modifications that were required for SOX9 binding. However, phosphorylation of H3S28 abolished SOX9 binding to histone tails in vitro and in vivo. Interestingly, we observed increased enrichment of H3S28P at sites where SOX9 opened chromatin, suggesting that following chromatin opening, H3S28 is phosphorylated and SOX9 is evicted from the site. Another possibility could be that SOX9 is evicted from the nucleosome, but binds the accessible DNA region following chromatin opening, which is indicated by the smaller average fragment size for C2 regions in our EChO analysis.

To summarize, we propose a model for SOX9-induced EndMT whereby SOX9 binds silent chromatin either by transient scanning or by specific binding to regions containing a SOX dimer motif and an H2A.Z-containing nucleosome. In the latter regions, SOX9 opens the local chromatin structure and enriches active histone modifications. In most cases, following chromatin opening, SOX9 gets evicted from the nucleosomes, possibly due to H3S28P enrichment, but the chromatin regions remain open and activate associated genes essential for the mesenchymal cell transition. The requirement for SOX9 in regulating the chromatin landscape may differ between cellular systems. For instance, SOX9 may not be crucial for epigenetic changes during chondrogenesis (20). In gonadal cell fate transition, DMRT1 was shown to open chromatin to allow binding of SOX9, while SOX9 did not have strong pioneering abilities (74). Nevertheless, we show that in EndMT, SOX9 expression alone is sufficient for chromatin opening and cell fate transition. Furthermore, our analysis of single-cell data points to SOX9 as a driver of EndMT in atherosclerosis. Tissue damage may stimulate EndMT to give rise to fibroblasts during wound healing or fibrotic diseases and EndMT forms cancer-associated fibroblasts in the tumor microenvironment, which regulates disease progression (75). Thus, SOX9 may be a potential driver of disease mechanisms when aberrantly expressed in endothelial cells.

## DATA AVAILABILITY

High-throughput sequencing data is publicly available through GEO (accession numbers: GSE155282 and GSE155290). In addition, differentially expressed genes from the RNA-seq and the clusters from SOX9 CUT&RUN and ATAC-seq are provided as supplemental tables (Supplementary Tables S4 and S5).

## Supplementary Material

gkac652_Supplemental_Files
