# Supplementary material for: SOX9 reprograms endothelial cells by altering the chromatin landscape
Source: Nucleic Acids Res. 2022 Jul 29;50(15):8547–65. doi: 10.1093/nar/gkac652 (PMC9410909; doi:10.1093/nar/gkac652)
Supplement: gkac652_Supplemental_Files [file gkac652_supplemental_files.zip › Legends Table S4_Table S5.docx]

**Supplementary table legends *Table S4.****Differentially expressed genes between HUVECs transduced with SOX9 or empty vector. Log2 FPKM values and ratios are provided.*

***Table S5.****Genomic coordinates of SOX9 CUT&RUN clusters C1-C4 peak summits and ATAC-seq peak summits in regions with increased and decreased chromatin accessibility upon SOX9 expression.*
