# Supplementary material for: SOX9 reprograms endothelial cells by altering the chromatin landscape
Source: Nucleic Acids Res. 2022 Jul 29;50(15):8547–65. doi: 10.1093/nar/gkac652 (PMC9410909; doi:10.1093/nar/gkac652)
Supplement: gkac652_Supplemental_Files [file gkac652_supplemental_files.zip › Supplemental material.pdf]

Supplemental material

SOX9 reprograms endothelial cells by altering the chromatin landscape.

Bettina M. Fuglerud, Sibyl Drissler[#], Jeremy Lotto[#], Tabea L. Stephan, Avinash Thakur, Rebecca Cullum, Pamela A. Hoodless

# These two authors contributed equally.

Table S1.
List of genes presented in Fig. 2E and supporting references. "Endothelial" genes encode markers of endothelial cells or shown to be downregulated upon EndMT. "Mesenchymal" genes encode mesenchymal markers or shown to be upregulated upon EndMT/EMT. Only genes that were expressed in HUVECs with/without SOX9 expression are included.

**Endothelial**	**Reference**
ACE	(1)
ANTXR1	(2)
CAV1	(3)
CAV2	(4)
CD151	(5)
CD34	(6)
CD93	(7)
CDH5	(6)
COLEC12	(8)
CXADR	(9)
DCBLD2	(10)
ECSCR	(11)
EGFL7	(12)
EMCN	(13)
ENG	(14)
EPOR	(15)
ERG	(16)
ESAM	(17)
FABP5	(18)
FLI1	(19)
FLT1	(20)
FLT4	(21)
ICAM1	(22)
ICAM2	(23)
JUP	(24)
KDR	(25)
KLF4	(26)
KRT19	(27)
LMO2	(28)
LYVE1	(29)
MCAM	(30)
MTUS1	(31)
NOS3	(6)
PECAM1	(6)

PODXL	(32)
PROCR	(33)
S1PR1	(34)
S1PR3	(34)
SELE	(35)
SELP	(36)
SOX18	(37)
SOX7	(38)
STAB1	(39)
TEK	(6)
THBD	(40)
THSD1	(41)
THSD7A	(42)
TIE1	(43)
TNFRSF10A	(44)
TNFRSF10B	(45)
TP53	(46)
VCAM1	(47)
VWF	(6)

**Mesenchymal**	**Reference**
ACTA2	(48)
ADAM12	(49)
AHNAK	(50)
ALCAM	(51)
BAMBI	(52)
BMP2	(53)
CALD1	(54)
CD44	(55)
CDH11	(56)
CDH2	(57)
CNN1	(58)
COL12A1	(59)
COL14A1	(60)
COL1A1	(61)
COL1A2	(62)
COL3A1	(62)
COL5A1	(63)
COL5A2	(62)

COL6A1	(64)
COL6A2	(64)
COL6A3	(65)
COL8A1	(66)
COL9A3	(67)
CTGF	(68)
CXCR4	(69)
DDR2	(62)
DLC1	(62)
FAP	(62)
FBLN5	(62)
FBN1	(70)
FOXC1	(71)
HEY1	(72)
HEY2	(73)
HEYL	(74)
IGFBP3	(61)
ITGA5	(75)
ITGAV	(76)
JAG1	(77)
LEF1	(78)
LOXL1	(79)
MMP14	(80)
MMP16	(81)
MMP2	(82)
MSN	(83)
MYH9	(84)
NEXN	(85)
NID2	(61)
NOTCH1	(86)
NOTCH3	(87)
NT5E	(88)
P4HA1	(89)
PLAT	(90)
PLAU	(91)
PLAUR	(80)
POSTN	(62)
PRKCA	(61)
PTX3	(62)
RECK	(61)
S100A4	(92)

SERPINE1	(91)
SERPINE2	(93)
SLC22A4	(61)
SNAI1	(48)
SPARC	(94)
SPOCK1	(95)
SRF	(96)
SRGN	(61)
TAGLN	(48)
TGFB1	(97)
TGFB2	(98)
TGFBR1	(99)
TGFBR2	(100)
TPM1	(101)
TUBA1A	(61)
VCAN	(102)
VIM	(103)
WNT5A	(104)
ZEB1	(105)
ZEB2	(106)

Table S2.
List of ENCODE datasets (bigwig files) used in Fig. 7A.

HUVEC ChIP dataset	ENCODE Accession
H2A.Z	ENCSR000ASC
H3K4me3	ENCSR578QSO
H3K9me3	ENCSR000ATB
H3K36me3	ENCSR000ALC
H3K79me2	ENCSR000ASD
H3K20me1	ENCSR000ALF

Table S3.

Modifications on histone peptide array incubated with recombinant SOX9 protein and probed with SOX9 antibody ranked by specificity factor. Specificity factor = average intensity of spots on array that contain the modifications/average intensity of spots that do not contain the modification.

Rank	Modification	Specificity Factor
1	H2A K9ac	1.6467
2	H2A K13ac	1.6427
3	H4 R19me2s	1.5867
4	H2A K5ac	1.5642
5	H2A S1P	1.4261
6	H3 R2me2s	1.4108
7	H3 T3P	1.4074
8	H3 K4me2	1.4026
9	H3 K9me2	1.3528
10	H4 R17me2s	1.351
11	H3 R8me2s	1.3284
12	H3 K4ac	1.3051
13	H4 R24me2s	1.2468
14	H3 R2Citr	1.2423
15	H3 K4me1	1.2305
16	H3 K9me1	1.2296
17	H3 R2me2a	1.2278
18	H3 K9me3	1.22
19	H4 R24me2a	1.1906
20	H4 K20me1	1.1664
21	H3 K4me3	1.1574
22	H3 K36me2	1.1387
23	H3 R8Citr	1.0911
24	H3 R8me2a	1.0442
25	H4 R19me2a	1.0305
26	H4 K20me2	0.9905
27	H3 K27me2	0.9752
28	H3 T11P	0.9347
29	H4 R17me2a	0.9344
30	H3 K14ac	0.9067
31	H2b K5ac	0.8957
32	H4 K20me3	0.8821
33	H3 K9ac	0.8719
34	H2b K12ac	0.8462
35	H3 K36ac	0.8151
36	H3 R17me2a	0.8034
37	H3 R26me2s	0.7936
38	H3 S10P	0.7886
39	H2b S14P	0.781
40	H3 K27me1	0.7735
41	H4 K20ac	0.7388
42	H3 R26me2a	0.6951
43	H3 K27me3	0.617
44	H3 R17me2s	0.5649
45	H3 K36me3	0.4827
46	H2b K15ac	0.4483
47	H4 K16ac	0.4207
48	H3 K27ac	0.3809
49	H3 K18ac	0.3521
50	H3 K36me1	0.2108
51	H3 R26Citr	0.1789
52	H4 K12ac	0.1712
53	H3 R17Citr	0.1575
54	H4 S1P	0.011
55	H4 R3me2a	0.011
56	H4 R3me2s	0.006
57	H4 K8ac	0.0059
58	H4 K5ac	0.0032
59	H3 S28P	0

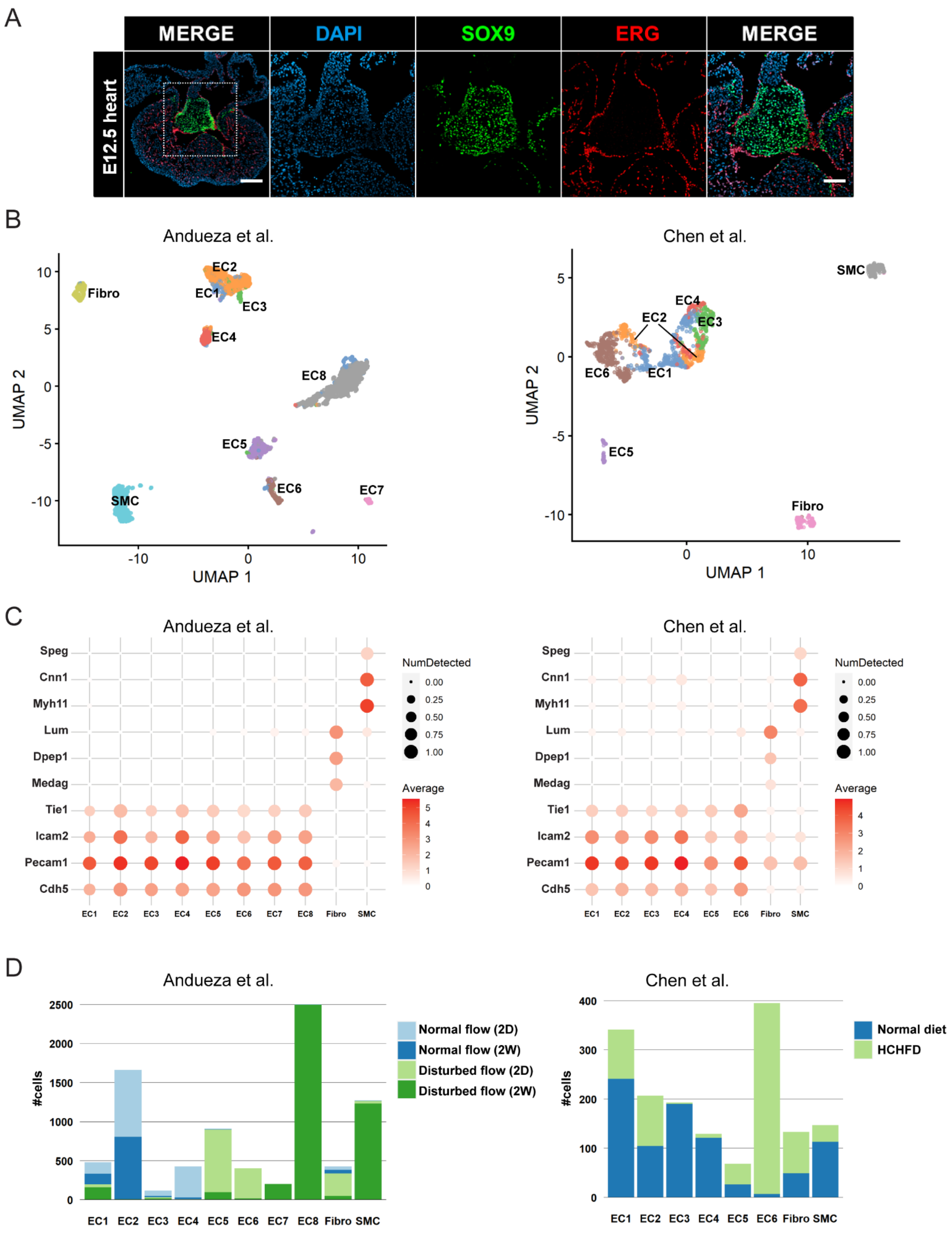
A
MERGE
DAPI
SOX9
ERG
MERGE
E12.5 heart
B
Andueza et al.
Chen et al.
Fibro
EC1
EC2
EC3
EC4
EC5
EC6
EC7
EC8
SMC
UMAP 1
UMAP 2
C
Andueza et al.
Chen et al.
Speg
Cnn1
Myh11
Lum
Dpep1
Medag
Tie1
Icam2
Pecam1
Cdh5
NumDetected
Average
D
Andueza et al.
Chen et al.
#cells
Normal flow (2D)
Normal flow (2W)
Disturbed flow (2D)
Disturbed flow (2W)
Normal diet
HCHFD

Figure S1.
A) SOX9 and ERG immunostaining of mouse embryonic hearts at E12.5. Scale bar whole hearts, 100 µm. Scale bar areas of interest, 25 µm. **B)** UMAP representation of scRNA-seq data of mouse atherosclerotic lesions from reanalyzed Andueza et al. (left) and Chen et al. (right). Cell populations include endothelial cells (Andueza et al.: EC1-EC8, Chen et al.: EC1-EC6), smooth muscle cells (SMC), and fibroblasts (Fibro). **C)** Dot plot displaying known marker genes for endothelial cells (EC), fibroblasts (Fibro), and smooth muscle cells (SMC). Size of the dot represents percentage of cells that express each gene. Color intensity indicates level of expression. **D)** Cell numbers in each cluster for the different conditions from Andueza et al. (left) and Chen et al. (right). 2D = cells isolated 2 days after carotid ligation, 2W = cells isolated 2 weeks after carotid ligation, HCHFD = mice on high cholesterol, high fat diet.

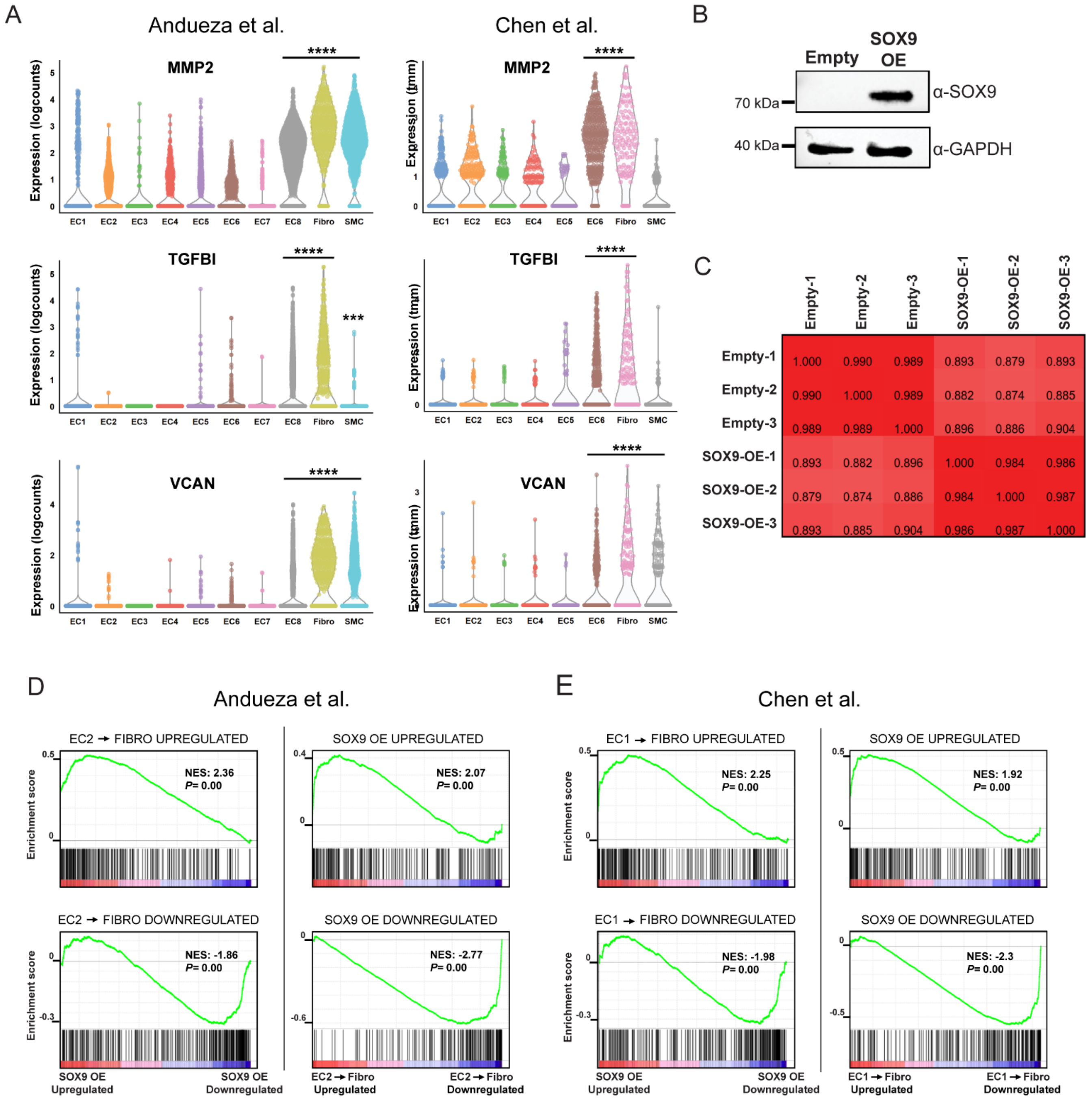

Figure S2.
A) MMP2, TGFBI, and VCAN expression levels in each cell cluster identified from scRNA-seq data from Andueza et al. (left) and Chen et al. (right). Significance was evaluated between clusters EC8, Fibro, or SMC and EC1-EC7 (Andueza et al.) and between EC6, Fibro, or SMC and EC1-EC6 (Chen et al.) by Wilcoxon rank sum tests with P-values (*P<0.05; **P<0.01; ***P<0.001; ****P<0.0001; ns P>0.05). **B)** Expression of SOX9 (SOX9 OE) in transduced HUVECs confirmed by western blotting. **C)** Pearson's correlation plot of FPKM counts visualizing the correlation (r) values between samples.

D) GSEA-based comparison of differential gene expression profile between HUVECs transduced with SOX9 or empty vector with top 500 upregulated or downregulated genes in Fibro cells from Andueza et al. (left), and of differential gene expression profile between EC2 and Fibro cells from Andueza et al. with top 500 upregulated or downregulated genes in SOX9-expressing HUVECs (right). **E)** GSEA-based comparison of differential gene expression profile between HUVECs transduced with SOX9 or empty vector with top 500 upregulated or downregulated genes in Fibro cells from Chen et al. (left), and of differential gene expression profile between EC1 and Fibro cells from Chen et al. with top 500 upregulated or downregulated genes in SOX9-expressing HUVECs (right).

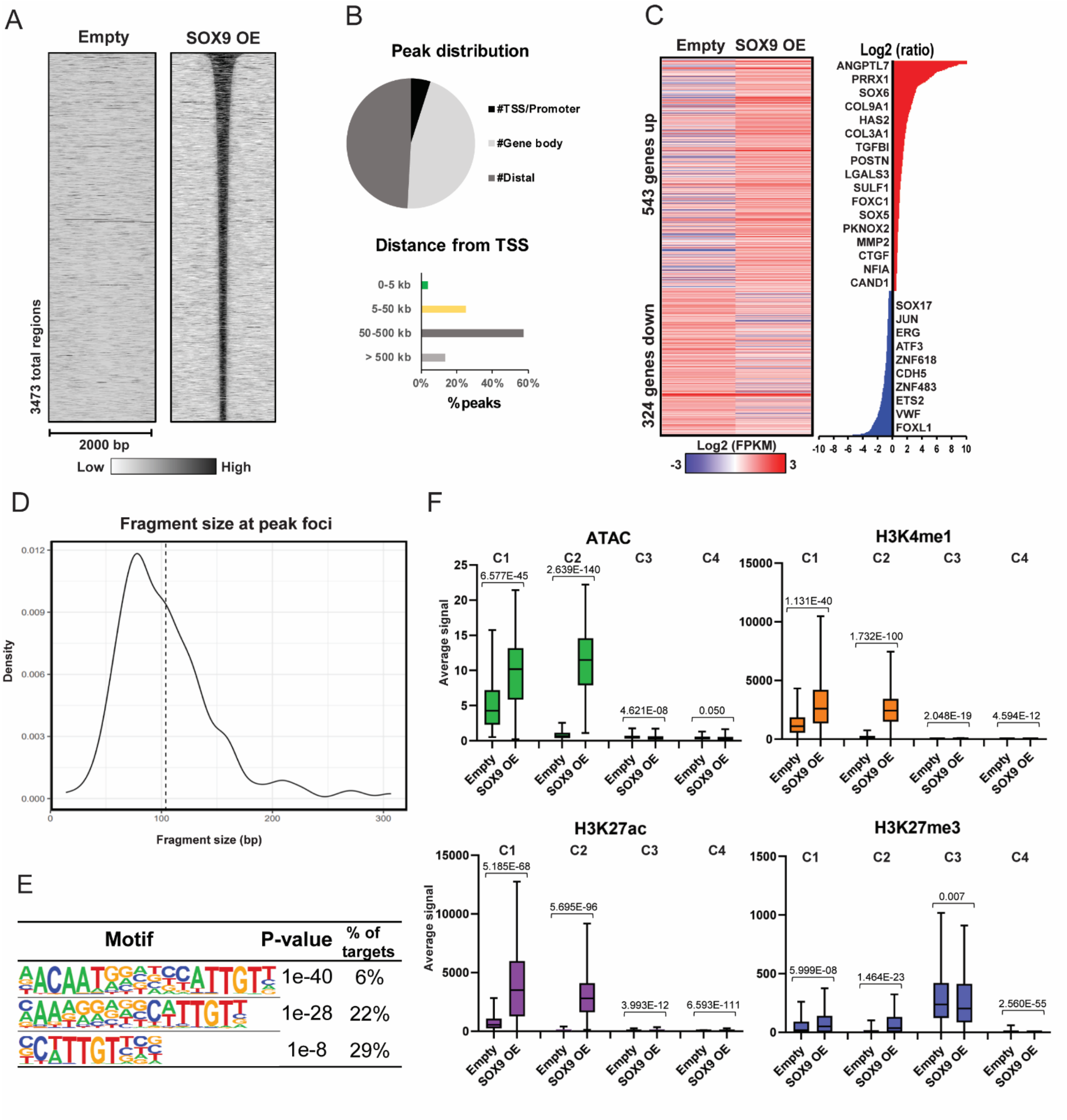

Figure S3.

A) Heatmap displaying SOX9 CUT&RUN signal within a 2 kb window around the summit of SOX9 bound regions. **B)** Distribution of SOX9 bound regions between TSS/promoter regions (5000 bp upstream to 500 bp downstream of TSS), within gene bodies, or distal. Lower panel shows distance from SOX9 bound region and the TSS of the associated genes. **C)** Heatmap of differentially expressed genes between cells transduced with SOX9 or empty vector that also contain an associated SOX9 bound region (direct SOX9 target genes). Heatmap shows log2 FPKM values while log2 ratios are displayed on the right with select genes highlighted. **D)** Density plot displaying distributions of average

fragment size at detected foci for SOX9 CUT&RUN. **E)** Top three motifs (based on P-value) identified in SOX9 bound regions. **F)** Average ATAC, H3K4me1, H3K27ac, and H3K27me3 signal at the summits of SOX9 bound regions in C1-C4. Significance was evaluated as in Fig. 1E. Mean is indicated with black line.

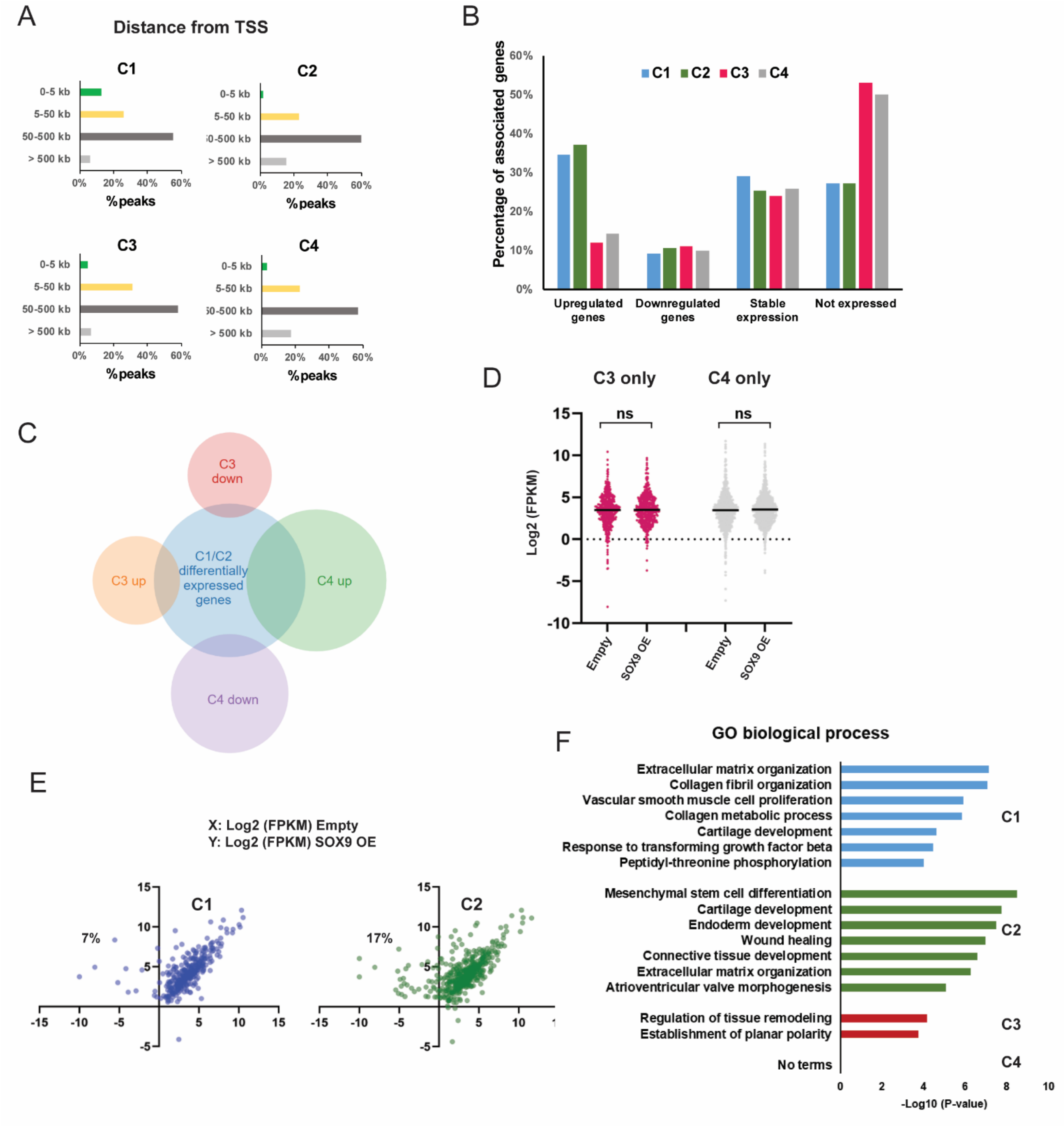

Figure S4:

A) Distance from SOX9 bound region and the TSS of the associated genes in C1-C4. **B)** Percentage of genes associated with SOX9 bound regions in C1-C4 that were upregulated, downregulated, unchanged (stable expression), or not expressed in HUVECs transduced with SOX9. **C)** Venn diagram showing the number of genes associated with SOX9 bound regions in cluster C3 and C4 that were upregulated or downregulated and their overlap with genes differentially expressed and associated with a SOX9 bound region in cluster C1 or C2. **D)** Scatterplots of log2 FPKM values of expressed genes with an associated SOX9 bound region in C3 or C4 which does not overlap with genes in C1 or C2.

Significance was evaluated as in Fig. 1E. Mean is indicated with black line. **E)** Scatterplot comparing log2 FPKM values between cells transduced with empty control vector (X-axis) or SOX9 (Y-axis) for all expressed genes in cluster C1 and C2. The percentage of genes silent in HUVECs transduced with empty vector that were expressed upon SOX9 expression is indicated. **F)** Top enriched (or the only enriched for C3) biological processes for genes associated with SOX9 bound regions in C1-C4.

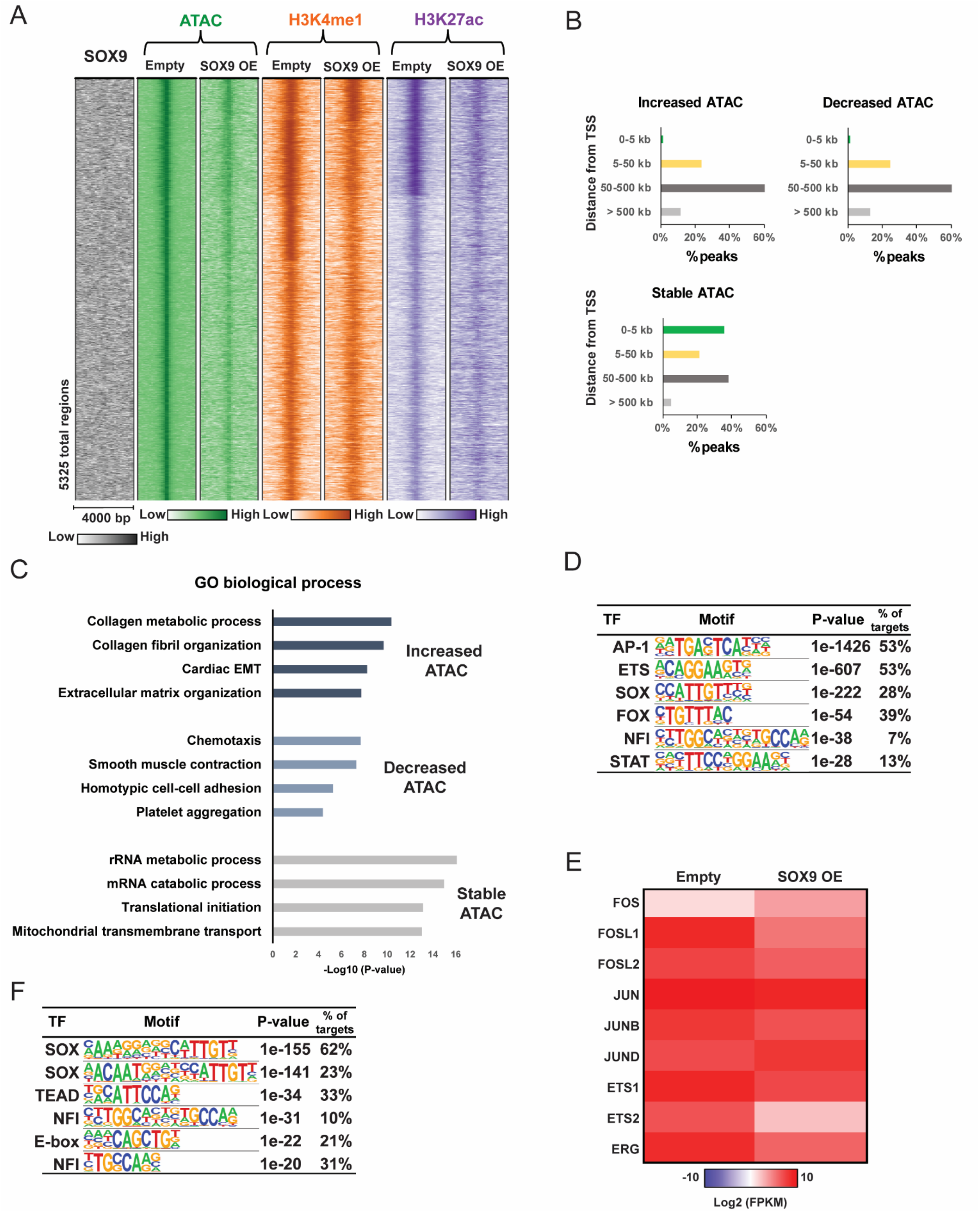

Figure S5.

A) Heatmap displaying SOX9 CUT&RUN, ATAC, and H3K4me1, and H3K27ac CUT&Tag signal

within a 4 kb window around the summit of decreased ATAC peaks upon SOX9 expression. **B)** Distance from regions with increased, decreased, or unchanged (stable) chromatin accessibility and the TSS of the associated genes. **C)** Top enriched biological processes for genes associated with increased, decreased, or unchanged chromatin accessibility regions. **D)** Top TF motifs enriched in regions with decreased chromatin accessibility. **E)** Heatmap displaying log2 FPKM values of expressed TFs with top two identified motifs in peaks with decreased chromatin accessibility. **F)** Top TF motifs enriched in regions with increased chromatin accessibility, but without detected SOX9 CUT&RUN signal at any of the given timepoints in Fig. 6D.

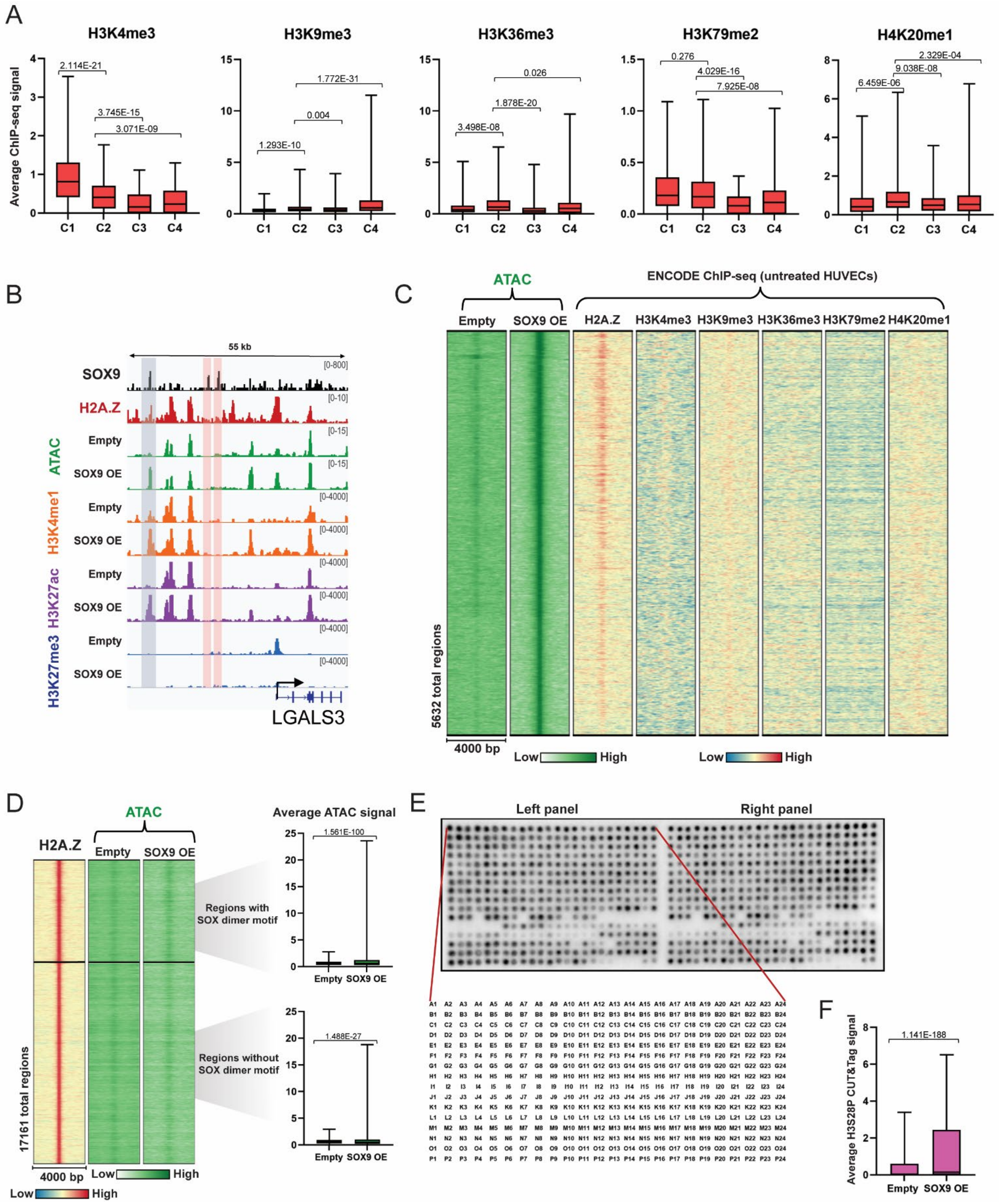

Figure S6.

A) Average H3K4me3, H3K9me3, H3K36me3, H3K79me2, and H3K20me1 ChIP-seq signal (ENCODE) at the summits of SOX9 bound regions in C1-C4. Significance was evaluated as in Fig. 1E. Mean is indicated with black line. **B)** Representative locus in cluster C2. Region with SOX9 binding,

chromatin opening, and H2A.Z enrichment is highlighted with gray box. Regions with SOX9 binding, but no chromatin opening, are highlighted with red boxes. **C)** Heatmap displaying ATAC, H2A.Z (ENCODE), H3K4me3 (ENCODE), H3K9me3 (ENCODE), H3K36me3 (ENCODE), H3K79me2 (ENCODE), and H4K20me1 (ENCODE) signal within a 4 kb window around the summit of ATAC peaks in regions with increased chromatin accessibility. **D)** Left panel: heatmap displaying H2A.Z (ENCODE) and ATAC signal indicated within a 4 kb window around the summit of H2A.Z peaks. Regions were divided into regions with SOX motifs (monomer and/or dimer) and regions without SOX motifs. The right panel displays the average ATAC signal in H2A.Z regions with SOX motifs or without SOX motifs. **E)** Histone peptide array containing 384 different histone tail modification combinations in duplicate (left panel and right panel) incubated with recombinant SOX9 protein and detected with anti-SOX9 primary antibody. The reference grid for histone peptide locations is shown. A full overview of the peptide spot positions can be downloaded from Active Motif's website at www.activemotif.com/modified. **F)** Average H3S28P signal at the summit of ATAC peaks in regions with increased chromatin accessibility.

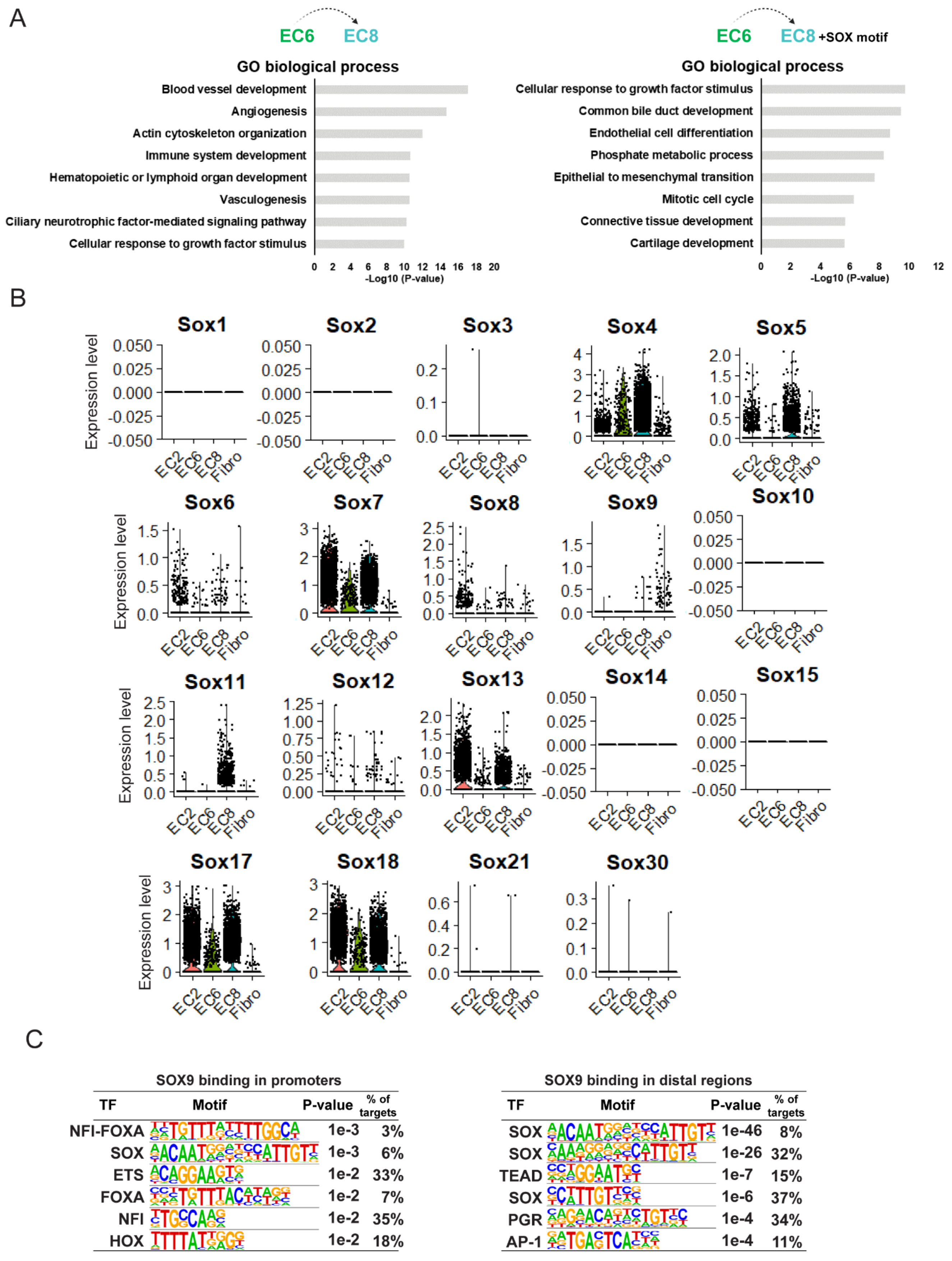
A
EC6
EC8
GO biological process
Blood vessel development
Angiogenesis
Actin cytoskeleton organization
Immune system development
Hematopoietic or lymphoid organ development
Vasculogenesis
Ciliary neurotrophic factor-mediated signaling pathway
Cellular response to growth factor stimulus
-Log10 (P-value)
EC6
EC8 +SOX motif
GO biological process
Cellular response to growth factor stimulus
Common bile duct development
Endothelial cell differentiation
Phosphate metabolic process
Epithelial to mesenchymal transition
Mitotic cell cycle
Connective tissue development
Cartilage development
-Log10 (P-value)
B
Sox1
Sox2
Sox3
Sox4
Sox5
Sox6
Sox7
Sox8
Sox9
Sox10
Sox11
Sox12
Sox13
Sox14
Sox15
Sox17
Sox18
Sox21
Sox30
Expression level
EC2
EC6
EC8
Fibro
C
SOX9 binding in promoters
TF
Motif
P-value
% of targets
NFI-FOXA 1e-3 3%
SOX 1e-3 6%
ETS 1e-2 33%
FOXA 1e-2 7%
NFI 1e-2 35%
HOX 1e-2 18%
SOX9 binding in distal regions
TF
Motif
P-value
% of targets
SOX 1e-46 8%
SOX 1e-26 32%
TEAD 1e-7 15%
SOX 1e-6 37%
PGR 1e-4 34%
AP-1 1e-4 11%

Figure S7.
A) Top enriched biological processes for genes associated with regions with increased chromatin accessibility between EC6 to EC8 (left) and in regions with increased chromatin accessibility between EC6 to EC8 that contain a SOX motif (right). **B)** Expression pattern of all SOX factors in the scRNA-seq clusters from Andueza et al. **C)** Top TF motifs enriched in SOX9 bound regions in promoters (left) or distal regions (right).

Supplemental Materials and Methods

Lentiviral vector production.
Lentivirus empty vector pRRL-cPPT/CTS-MNDU3-PGK-GFP-WPRE (107) and packaging/envelope vectors pCMV-dR8.74 (22036, Addgene), pCMV-VSV-G (8454, Addgene), and pRSV-Rev (12253, Addgene) were kindly provided by Dr. Andrew Weng (Terry Fox Laboratory, BC Cancer). SOX9 human cDNA was kindly provided by William Stanford (Sprott Centre for Stem Cell Research, Ottawa Hospital Research Institute) and was cloned into pRRL-cPPT/CTS-MNDU3-PGK-GFP-WPRE immediately downstream of the MINDU3 promoter. The construct was verified by sequencing. For lentivirus packaging, HEK293T (CRL-3216, ATCC) cells were cultured in Dulbecco's modified Eagle's medium (DMEM) supplemented with 10% fetal bovine serum (FBS). The lentivirus vector and packaging plasmids were cotransfected using polyethyleneimine. Lentiviral supernatants were collected 48 hours after transfection.

Protein extraction and western blotting.
SDS loading dye (50 mM Tris-HCl pH 6.8, 2% SDS, 10% glycerol, 12.5 mM EDTA, 0.02% bromophenol blue, 10% beta-mercaptoethanol) was added to pelleted cells and the lysates were sonicated before loaded onto an SDS-polyacrylamide gel and transferred onto a PVDF membrane. The membranes were blocked using 3% non-fat milk in TBS. After blocking, the membranes were incubated overnight at 4 °C with primary antibody. The membranes were then incubated with HRP-conjugated secondary antibody for 1 hour at room temperature. HRP activity was detected with Pierce ECL Western Blotting Substrate (Thermo Fisher Scientific) and imaged using ChemiDoc Imaging System (Bio-Rad). Antibodies against the following proteins were applied: SOX9 (1:2000, AB5535, Millipore) and GAPDH (1:4000, AM4300, Thermo Fisher Scientific).

RNA isolation, reverse transcription and RT-qPCR.
RNA was extracted using TRIzol (Thermo Fisher Scientific). 500 ng RNA was used for reverse transcription with First Strand cDNA Synthesis Kit (Roche). RT-qPCR was performed using FastStart Universal SYBR Green Master Mix (Roche) in a StepOnePlus Real-Time PCR System (Applied Biosystems). The values of RNA expression were normalized to the relative amount of the reference gene *GAPDH.* Primer sequences (5'- 3'):

Human GAPDH Forward: GGTGTCGCTGAAGTCAGAG
Human GAPDH Reverse: GGACCTGACCTGCCGTCTAGAA
Human SOX9 Forward: CACGGAGCAGACGCACATCT
Human SOX9 Reverse: TCTCGCTTCAGGTCAGCCTT

Supplemental References.

1. Nagai,T., Kanasaki,M., Srivastava,S.P., Nakamura,Y., Ishigaki,Y., Kitada,M., Shi,S., Kanasaki,K. and Koya,D. (2014) N-acetyl-seryl-aspartyl-lysyl-proline inhibits diabetes-associated kidney fibrosis and endothelial-mesenchymal transition. *Biomed Res. Int.*, **2014**, 696475.

2. Carson-Walter,E.B., Watkins,D.N., Nanda,A., Vogelstein,B., Kinzler,K.W. and St Croix,B. (2001) Cell surface tumor endothelial markers are conserved in mice and humans. *Cancer Res.*, **61**, 6649–6655.

3. Bauer,P.M., Yu,J., Chen,Y., Hickey,R., Bernatchez,P.N., Looft-Wilson,R., Huang,Y., Giordano,F., Stan,R. V and Sessa,W.C. (2005) Endothelial-specific expression of caveolin-1 impairs microvascular permeability and angiogenesis. *Proc. Natl. Acad. Sci. U. S. A.*, **102**, 204–209.

4. Dragoi,A.-M., Swiss,R., Gao,B. and Agaisse,H. (2014) Novel strategies to enforce an epithelial phenotype in mesenchymal cells. *Cancer Res.*, **74**, 3659–3672.

5. Zhang,F., Michaelson,J.E., Moshiach,S., Sachs,N., Zhao,W., Sun,Y., Sonnenberg,A., Lahti,J.M., Huang,H. and Zhang,X.A. (2011) Tetraspanin CD151 maintains vascular stability by balancing the forces of cell adhesion and cytoskeletal tension. *Blood*, **118**, 4274–4284.

6. Pereira,C.-F., Chang,B., Qiu,J., Niu,X., Papatsenko,D., Hendry,C.E., Clark,N.R., Nomura-Kitabayashi,A., Kovacic,J.C., Ma'ayan,A., *et al.* (2013) Induction of a hemogenic program in mouse fibroblasts. *Cell Stem Cell*, **13**, 205–218.

7. Fonseca,M.I., Carpenter,P.M., Park,M., Palmarini,G., Nelson,E.L. and Tenner,A.J. (2001) C1qR(P), a myeloid cell receptor in blood, is predominantly expressed on endothelial cells in human tissue. *J. Leukoc. Biol.*, **70**, 793–800.

8. Ohtani,K., Suzuki,Y., Eda,S., Kawai,T., Kase,T., Keshi,H., Sakai,Y., Fukuoh,A., Sakamoto,T., Itabe,H., *et al.* (2001) The membrane-type collectin CL-P1 is a scavenger receptor on vascular endothelial cells. *J. Biol. Chem.*, **276**, 44222–44228.

9. Lacher,M.D., Tiirikainen,M.I., Saunier,E.F., Christian,C., Anders,M., Oft,M., Balmain,A., Akhurst,R.J. and Korn,W.M. (2006) Transforming growth factor-beta receptor inhibition enhances adenoviral infectability of carcinoma cells via up-regulation of Coxsackie and Adenovirus Receptor in conjunction with reversal of epithelial-mesenchymal transition. *Cancer Res.*, **66**, 1648–1657.

10. Nie,L., Guo,X., Esmailzadeh,L., Zhang,J., Asadi,A., Collinge,M., Li,X., Kim,J.-D., Woolls,M., Jin,S.-W., *et al.* (2013) Transmembrane protein ESDN promotes endothelial VEGF signaling and regulates angiogenesis. *J. Clin. Invest.*, **123**, 5082–5097.

11. Armstrong,L.-J., Heath,V.L., Sanderson,S., Kaur,S., Beesley,J.F.J., Herbert,J.M.J., Legg,J.A., Poulsom,R. and Bicknell,R. (2008) ECSM2, an endothelial specific filamin a binding protein that mediates chemotaxis. *Arterioscler. Thromb. Vasc. Biol.*, **28**, 1640–1646.

12. Parker,L.H., Schmidt,M., Jin,S.-W., Gray,A.M., Beis,D., Pham,T., Frantz,G., Palmieri,S., Hillan,K., Stainier,D.Y.R., *et al.* (2004) The endothelial-cell-derived secreted factor Egfl7 regulates vascular tube formation. *Nature*, **428**, 754–758.

13. Liu,C., Shao,Z.M., Zhang,L., Beatty,P., Sartippour,M., Lane,T., Livingston,E. and Nguyen,M. (2001) Human endomucin is an endothelial marker. *Biochem. Biophys. Res. Commun.*, **288**, 129–136.

14. Cheifetz,S., Bellón,T., Calés,C., Vera,S., Bernabeu,C., Massagué,J. and Letarte,M. (1992) Endoglin is a component of the transforming growth factor-beta receptor system in human endothelial cells. *J. Biol. Chem.*, **267**, 19027–19030.

15. Anagnostou,A., Liu,Z., Steiner,M., Chin,K., Lee,E.S., Kessimian,N. and Noguchi,C.T. (1994) Erythropoietin receptor mRNA expression in human endothelial cells. *Proc. Natl. Acad. Sci. U. S. A.*, **91**, 3974–3978.

16. Nikolova-Krstevski,V., Yuan,L., Le Bras,A., Vijayaraj,P., Kondo,M., Gebauer,I., Bhasin,M., Carman,C. V and Oettgen,P. (2009) ERG is required for the differentiation of embryonic stem cells along the endothelial lineage. *BMC Dev. Biol.*, **9**, 72.

17. Elcheva,I., Brok-Volchanskaya,V., Kumar,A., Liu,P., Lee,J.-H., Tong,L., Vodyanik,M., Swanson,S., Stewart,R., Kyba,M., *et al.* (2014) Direct induction of haematoendothelial programs in human pluripotent stem cells by transcriptional regulators. *Nat. Commun.*, **5**, 4372.

18. Masouyé,I., Hagens,G., Van Kuppevelt,T.H., Madsen,P., Saurat,J.H., Veerkamp,J.H., Pepper,M.S. and Siegenthaler,G. (1997) Endothelial cells of the human microvasculature express epidermal fatty acid-binding protein. *Circ. Res.*, **81**, 297–303.

19. Asano,Y., Stawski,L., Hant,F., Highland,K., Silver,R., Szalai,G., Watson,D.K. and Trojanowska,M. (2010) Endothelial Fli1 deficiency impairs vascular homeostasis: a role in scleroderma vasculopathy. *Am. J. Pathol.*, **176**, 1983–1998.

20. Seetharam,L., Gotoh,N., Maru,Y., Neufeld,G., Yamaguchi,S. and Shibuya,M. (1995) A unique signal transduction from FLT tyrosine kinase, a receptor for vascular endothelial growth factor VEGF. *Oncogene*, **10**, 135–147.

21. Kaipainen,A., Korhonen,J., Pajusola,K., Aprelikova,O., Persico,M.G., Terman,B.I. and Alitalo,K. (1993) The related FLT4, FLT1, and KDR receptor tyrosine kinases show distinct expression patterns in human fetal endothelial cells. *J. Exp. Med.*, **178**, 2077–2088.

22. Almenar-Queralt,A., Duperray,A., Miles,L.A., Felez,J. and Altieri,D.C. (1995) Apical topography and modulation of ICAM-1 expression on activated endothelium. *Am. J. Pathol.*, **147**, 1278–1288.

23. Cowan,P.J., Tsang,D., Pedic,C.M., Abbott,L.R., Shinkel,T.A., d'Apice,A.J. and Pearse,M.J. (1998) The human ICAM-2 promoter is endothelial cell-specific in vitro and in vivo and contains critical Sp1 and GATA binding sites. *J. Biol. Chem.*, **273**, 11737–11744.

24. Holen,I., Whitworth,J., Nutter,F., Evans,A., Brown,H.K., Lefley,D. V, Barbaric,I., Jones,M. and Ottewell,P.D. (2012) Loss of plakoglobin promotes decreased cell-cell contact, increased invasion, and breast cancer cell dissemination in vivo. *Breast Cancer Res.*, **14**, R86.

25. Terman,B.I., Carrion,M.E., Kovacs,E., Rasmussen,B.A., Eddy,R.L. and Shows,T.B. (1991) Identification of a new endothelial cell growth factor receptor tyrosine kinase. *Oncogene*, **6**, 1677–1683.

26. Sangwung,P., Zhou,G., Nayak,L., Chan,E.R., Kumar,S., Kang,D.-W., Zhang,R., Liao,X., Lu,Y., Sugi,K., *et al.* (2017) KLF2 and KLF4 control endothelial identity and vascular integrity. *JCI insight*, **2**, e91700.

27. Loriot,C., Burnichon,N., Gadessaud,N., Vescovo,L., Amar,L., Libé,R., Bertherat,J., Plouin,P.-F., Jeunemaitre,X., Gimenez-Roqueplo,A.-P., *et al.* (2012) Epithelial to mesenchymal transition is activated in metastatic pheochromocytomas and paragangliomas caused by SDHB gene mutations. *J. Clin. Endocrinol. Metab.*, **97**, E954-62.

28. Gratzinger,D., Zhao,S., West,R., Rouse,R. V, Vogel,H., Gil,E.C., Levy,R., Lossos,I.S. and Natkunam,Y. (2009) The transcription factor LMO2 is a robust marker of vascular endothelium and vascular neoplasms and selected other entities. *Am. J. Clin. Pathol.*, **131**, 264–278.

29. Gordon,E.J., Gale,N.W. and Harvey,N.L. (2008) Expression of the hyaluronan receptor LYVE-1 is not restricted to the lymphatic vasculature; LYVE-1 is also expressed on embryonic blood vessels. *Dev. Dyn. an Off. Publ. Am. Assoc. Anat.*, **237**, 1901–1909.

30. Schrage,A., Loddenkemper,C., Erben,U., Lauer,U., Hausdorf,G., Jungblut,P.R., Johnson,J., Knolle,P.A., Zeitz,M., Hamann,A., *et al.* (2008) Murine CD146 is widely expressed on endothelial cells and is recognized by the monoclonal antibody ME-9F1. *Histochem. Cell Biol.*, **129**, 441–451.

31. Zhao,T., Ding,X., Chang,B., Zhou,X. and Wang,A. (2015) MTUS1/ATIP3a down-regulation is associated with enhanced migration, invasion and poor prognosis in salivary adenoid cystic carcinoma. *BMC Cancer*, **15**, 203.

32. Horvat,R., Hovorka,A., Dekan,G., Poczewski,H. and Kerjaschki,D. (1986) Endothelial cell membranes contain podocalyxin--the major sialoprotein of visceral glomerular epithelial cells. *J. Cell Biol.*, **102**, 484–491.

33. Fukudome,K. and Esmon,C.T. (1994) Identification, cloning, and regulation of a novel endothelial cell protein C/activated protein C receptor. *J. Biol. Chem.*, **269**, 26486–26491.

34. Kimura,T., Watanabe,T., Sato,K., Kon,J., Tomura,H., Tamama,K., Kuwabara,A., Kanda,T., Kobayashi,I., Ohta,H., *et al.* (2000) Sphingosine 1-phosphate stimulates proliferation and migration of human endothelial cells possibly through the lipid receptors, Edg-1 and Edg-3. *Biochem. J.*, **348 Pt 1**, 71–76.

35. Collins,T., Williams,A., Johnston,G.I., Kim,J., Eddy,R., Shows,T., Gimbrone,M.A.J. and Bevilacqua,M.P. (1991) Structure and chromosomal location of the gene for endothelial-leukocyte adhesion molecule 1. *J. Biol. Chem.*, **266**, 2466–2473.

36. Polley,M.J., Phillips,M.L., Wayner,E., Nudelman,E., Singhal,A.K., Hakomori,S. and Paulson,J.C. (1991) CD62 and endothelial cell-leukocyte adhesion molecule 1 (ELAM-1) recognize the same carbohydrate ligand, sialyl-Lewis x. *Proc. Natl. Acad. Sci. U. S. A.*, **88**, 6224–6228.

37. Hosking,B.M., Wang,S.C., Chen,S.L., Penning,S., Koopman,P. and Muscat,G.E. (2001) SOX18 directly interacts with MEF2C in endothelial cells. *Biochem. Biophys. Res. Commun.*, **287**, 493–500.

38. Behrens,A.N., Zierold,C., Shi,X., Ren,Y., Koyano-Nakagawa,N., Garry,D.J. and Martin,C.M. (2014) Sox7 is regulated by ETV2 during cardiovascular development. *Stem Cells Dev.*, **23**, 2004–2013.

39. Kzhyshkowska,J. (2010) Multifunctional receptor stabilin-1 in homeostasis and disease. *ScientificWorldJournal.*, **10**, 2039–2053.

40. Sadler,J.E. (1997) Thrombomodulin structure and function. *Thromb. Haemost.*, **78**, 392–395.

41. Haasdijk,R.A., Den Dekker,W.K., Cheng,C., Tempel,D., Szulcek,R., Bos,F.L., Hermkens,D.M.A., Chrifi,I., Brandt,M.M., Van Dijk,C., *et al.* (2016) THSD1 preserves vascular integrity and protects against intraplaque haemorrhaging in ApoE-/- mice. *Cardiovasc. Res.*, **110**, 129–139.

42. Wang,C.-H., Su,P.-T., Du,X.-Y., Kuo,M.-W., Lin,C.-Y., Yang,C.-C., Chan,H.-S., Chang,S.-J., Kuo,C., Seo,K., *et al.* (2010) Thrombospondin type I domain containing 7A (THSD7A) mediates endothelial cell migration and tube formation. *J. Cell. Physiol.*, **222**, 685–694.

43. Partanen,J., Armstrong,E., Mäkelä,T.P., Korhonen,J., Sandberg,M., Renkonen,R., Knuutila,S., Huebner,K. and Alitalo,K. (1992) A novel endothelial cell surface receptor tyrosine kinase with extracellular epidermal growth factor homology domains. *Mol. Cell. Biol.*, **12**, 1698–1707.

44. Li,J.H., Kirkiles-Smith,N.C., McNiff,J.M. and Pober,J.S. (2003) TRAIL induces apoptosis and inflammatory gene expression in human endothelial cells. *J. Immunol.*, **171**, 1526–1533.

45. Perrot-Applanat,M., Vacher,S., Toullec,A., Pelaez,I., Velasco,G., Cormier,F., Saad,H.E.S., Lidereau,R., Baud,V. and Bièche,I. (2011) Similar NF-κB gene signatures in TNF-α treated human endothelial cells and breast tumor biopsies. *PLoS One*, **6**, e21589.

46. Ghosh,A.K., Nagpal,V., Covington,J.W., Michaels,M.A. and Vaughan,D.E. (2012) Molecular basis of cardiac endothelial-to-mesenchymal transition (EndMT): differential expression of microRNAs during EndMT. *Cell. Signal.*, **24**, 1031–1036.

47. Cybulsky,M.I. and Gimbrone,M.A.J. (1991) Endothelial expression of a mononuclear leukocyte adhesion molecule during atherogenesis. *Science*, **251**, 788–791.

48. Cooley,B.C., Nevado,J., Mellad,J., Yang,D., St Hilaire,C., Negro,A., Fang,F., Chen,G., San,H., Walts,A.D., *et al.* (2014) TGF-β signaling mediates endothelial-to-mesenchymal transition (EndMT) during vein graft remodeling. *Sci. Transl. Med.*, **6**, 227ra34.

49. Buenrostro,J.D., Wu,B., Chang,H.Y. and Greenleaf,W.J. (2015) ATAC-seq: A Method for Assaying Chromatin Accessibility Genome-Wide. *Curr. Protoc. Mol. Biol.*, **109**, 21.29.1-21.29.9.

50. Shankar,J., Messenberg,A., Chan,J., Underhill,T.M., Foster,L.J. and Nabi,I.R. (2010) Pseudopodial actin dynamics control epithelial-mesenchymal transition in metastatic cancer cells. *Cancer Res.*, **70**, 3780–3790.

51. Fujiwara,K., Ohuchida,K., Sada,M., Horioka,K., Ulrich,C.D. 3rd, Shindo,K., Ohtsuka,T., Takahata,S., Mizumoto,K., Oda,Y., *et al.* (2014) CD166/ALCAM expression is characteristic of tumorigenicity and invasive and migratory activities of pancreatic cancer cells. *PLoS One*, **9**, e107247.

52. Fritzmann,J., Morkel,M., Besser,D., Budczies,J., Kosel,F., Brembeck,F.H., Stein,U., Fichtner,I., Schlag,P.M. and Birchmeier,W. (2009) A colorectal cancer expression profile that includes transforming growth factor beta inhibitor BAMBI predicts metastatic potential. *Gastroenterology*, **137**, 165–175.

53. Ma,L., Lu,M.-F., Schwartz,R.J. and Martin,J.F. (2005) Bmp2 is essential for cardiac cushion epithelial-mesenchymal transition and myocardial patterning. *Development*, **132**, 5601–5611.

54. Morita,T., Mayanagi,T. and Sobue,K. (2007) Dual roles of myocardin-related transcription factors in epithelial mesenchymal transition via slug induction and actin remodeling. *J. Cell Biol.*, **179**, 1027–1042.

55. Cho,S.H., Park,Y.S., Kim,H.J., Kim,C.H., Lim,S.W., Huh,J.W., Lee,J.H. and Kim,H.R. (2012) CD44 enhances the epithelial-mesenchymal transition in association with colon cancer invasion. *Int. J. Oncol.*, **41**, 211–218.

56. Schneider,D.J., Wu,M., Le,T.T., Cho,S.-H., Brenner,M.B., Blackburn,M.R. and Agarwal,S.K. (2012) Cadherin-11 contributes to pulmonary fibrosis: potential role in TGF-β production and epithelial to mesenchymal transition. *FASEB J. Off. Publ. Fed. Am. Soc. Exp. Biol.*, **26**, 503–512.

57. Wang,M., Ren,D., Guo,W., Huang,S., Wang,Z., Li,Q., Du,H., Song,L. and Peng,X. (2016) N-cadherin promotes epithelial-mesenchymal transition and cancer stem cell-like traits via ErbB signaling in prostate cancer cells. *Int. J. Oncol.*, **48**, 595–606.

58. Haque,I., Mehta,S., Majumder,M., Dhar,K., De,A., McGregor,D., Van Veldhuizen,P.J., Banerjee,S.K. and Banerjee,S. (2011) Cyr61/CCN1 signaling is critical for epithelial-mesenchymal transition and stemness and promotes pancreatic carcinogenesis. *Mol. Cancer*, **10**, 8.

59. Xiang,Z., Li,J., Song,S., Wang,J., Cai,W., Hu,W., Ji,J., Zhu,Z., Zang,L., Yan,R., *et al.* (2019) A positive feedback between IDO1 metabolite and COL12A1 via MAPK pathway to promote gastric cancer metastasis. *J. Exp. Clin. Cancer Res.*, **38**, 314.

60. Aoyagi,K., Minashi,K., Igaki,H., Tachimori,Y., Nishimura,T., Hokamura,N., Ashida,A., Daiko,H., Ochiai,A., Muto,M., *et al.* (2011) Artificially induced epithelial-mesenchymal transition in surgical subjects: its implications in clinical and basic cancer research. *PLoS One*, **6**, e18196.

61. Gröger,C.J., Grubinger,M., Waldhör,T., Vierlinger,K. and Mikulits,W. (2012) Meta-analysis of gene expression signatures defining the epithelial to mesenchymal transition during cancer progression. *PLoS One*, **7**, e51136.

62. Taube,J.H., Herschkowitz,J.I., Komurov,K., Zhou,A.Y., Gupta,S., Yang,J., Hartwell,K., Onder,T.T., Gupta,P.B., Evans,K.W., *et al.* (2010) Core epithelial-to-mesenchymal transition interactome gene-expression signature is associated with claudin-low and metaplastic breast cancer subtypes. *Proc. Natl. Acad. Sci. U. S. A.*, **107**, 15449–15454.

63. Joseph,J. V, Conroy,S., Tomar,T., Eggens-Meijer,E., Bhat,K., Copray,S., Walenkamp,A.M.E., Boddeke,E., Balasubramanyian,V., Wagemakers,M., *et al.* (2014) TGF-β is an inducer of ZEB1-dependent mesenchymal transdifferentiation in glioblastoma that is associated with tumor invasion. *Cell Death Dis.*, **5**, e1443.

64. Jechlinger,M., Grunert,S., Tamir,I.H., Janda,E., Lüdemann,S., Waerner,T., Seither,P., Weith,A., Beug,H. and Kraut,N. (2003) Expression profiling of epithelial plasticity in tumor progression. *Oncogene*, **22**, 7155–7169.

65. Huang,Y., Li,G., Wang,K., Mu,Z., Xie,Q., Qu,H., Lv,H. and Hu,B. (2018) Collagen Type VI Alpha 3 Chain Promotes Epithelial-Mesenchymal Transition in Bladder Cancer Cells via Transforming Growth Factor β (TGF-β)/Smad Pathway. *Med. Sci. Monit. Int. Med. J. Exp. Clin. Res.*, **24**, 5346–5354.

66. Minafra,L., Bravatà,V., Forte,G.I., Cammarata,F.P., Gilardi,M.C. and Messa,C. (2014) Gene expression profiling of epithelial-mesenchymal transition in primary breast cancer cell culture. *Anticancer Res.*, **34**, 2173–2183.

67. Vrljicak,P., Cullum,R., Xu,E., Chang,A.C.Y., Wederell,E.D., Bilenky,M., Jones,S.J.M.,

Marra,M.A., Karsan,A. and Hoodless,P.A. (2012) Twist1 transcriptional targets in the developing atrio-ventricular canal of the mouse. *PLoS One*, **7**, e40815.

68. Shafieian,M., Chen,S. and Wu,S. (2015) Integrin-linked kinase mediates CTGF-induced epithelial to mesenchymal transition in alveolar type II epithelial cells. *Pediatr. Res.*, **77**, 520–527.

69. Li,X., Li,P., Chang,Y., Xu,Q., Wu,Z., Ma,Q. and Wang,Z. (2014) The SDF-1/CXCR4 axis induces epithelial–mesenchymal transition in hepatocellular carcinoma. *Mol. Cell. Biochem.*, **392**, 77–84.

70. Lien,H.-C., Lee,Y.-H., Juang,Y.-L. and Lu,Y.-T. (2019) Fibrillin-1, a novel TGF-beta-induced factor, is preferentially expressed in metaplastic carcinoma with spindle sarcomatous metaplasia. *Pathology*, **51**, 375–383.

71. Zhu,X., Wei,L., Bai,Y., Wu,S. and Han,S. (2017) FoxC1 promotes epithelial-mesenchymal transition through PBX1 dependent transactivation of ZEB2 in esophageal cancer. *Am. J. Cancer Res.*, **7**, 1642–1653.

72. Zavadil,J., Cermak,L., Soto-Nieves,N. and Böttinger,E.P. (2004) Integration of TGF-beta/Smad and Jagged1/Notch signalling in epithelial-to-mesenchymal transition. *EMBO J.*, **23**, 1155–1165.

73. Miao,L., Li,J., Li,J., Tian,X., Lu,Y., Hu,S., Shieh,D., Kanai,R., Zhou,B.-Y., Zhou,B., *et al.* (2018) Notch signaling regulates Hey2 expression in a spatiotemporal dependent manner during cardiac morphogenesis and trabecular specification. *Sci. Rep.*, **8**, 2678.

74. Bielesz,B., Sirin,Y., Si,H., Niranjan,T., Gruenwald,A., Ahn,S., Kato,H., Pullman,J., Gessler,M., Haase,V.H., *et al.* (2010) Epithelial Notch signaling regulates interstitial fibrosis development in the kidneys of mice and humans. *J. Clin. Invest.*, **120**, 4040–4054.

75. Qin,L., Chen,X., Wu,Y., Feng,Z., He,T., Wang,L., Liao,L. and Xu,J. (2011) Steroid receptor coactivator-1 upregulates integrin α_5 expression to promote breast cancer cell adhesion and migration. *Cancer Res.*, **71**, 1742–1751.

76. Wehbe,M., Soudja,S.M., Mas,A., Chasson,L., Guinamard,R., de Tenbossche,C.P., Verdeil,G., Van den Eynde,B. and Schmitt-Verhulst,A.-M. (2012) Epithelial-mesenchymal-transition-like and TGFβ pathways associated with autochthonous inflammatory melanoma development in mice. *PLoS One*, **7**, e49419.

77. Leong,K.G., Niessen,K., Kulic,I., Raouf,A., Eaves,C., Pollet,I. and Karsan,A. (2007) Jagged1-mediated Notch activation induces epithelial-to-mesenchymal transition through Slug-induced repression of E-cadherin. *J. Exp. Med.*, **204**, 2935–2948.

78. Kobayashi,W. and Ozawa,M. (2018) The epithelial-mesenchymal transition induced by transcription factor LEF-1 is independent of β-catenin. *Biochem. Biophys. reports*, **15**, 13–18.

79. Ji,H., Ramsey,M.R., Hayes,D.N., Fan,C., McNamara,K., Kozlowski,P., Torrice,C., Wu,M.C., Shimamura,T., Perera,S.A., *et al.* (2007) LKB1 modulates lung cancer differentiation and metastasis. *Nature*, **448**, 807–810.

80. Sarrió,D., Rodriguez-Pinilla,S.M., Hardisson,D., Cano,A., Moreno-Bueno,G. and Palacios,J. (2008) Epithelial-mesenchymal transition in breast cancer relates to the basal-like phenotype. *Cancer Res.*, **68**, 989–997.

81. Shen,Z., Wang,X., Yu,X., Zhang,Y. and Qin,L. (2017) MMP16 promotes tumor metastasis and indicates poor prognosis in hepatocellular carcinoma. *Oncotarget*, **8**, 72197–72204.

82. Wiercinska,E., Naber,H.P.H., Pardali,E., van der Pluijm,G., van Dam,H. and ten Dijke,P. (2011) The TGF-β/Smad pathway induces breast cancer cell invasion through the up-regulation of matrix metalloproteinase 2 and 9 in a spheroid invasion model system. *Breast Cancer Res. Treat.*, **128**, 657–666.

83. Haynes,J., Srivastava,J., Madson,N., Wittmann,T. and Barber,D.L. (2011) Dynamic actin remodeling during epithelial-mesenchymal transition depends on increased moesin expression. *Mol. Biol. Cell*, **22**, 4750–4764.

84. Beach,J.R., Hussey,G.S., Miller,T.E., Chaudhury,A., Patel,P., Monslow,J., Zheng,Q., Keri,R.A., Reizes,O., Bresnick,A.R., *et al.* (2011) Myosin II isoform switching mediates invasiveness after TGF-β-induced epithelial-mesenchymal transition. *Proc. Natl. Acad. Sci. U. S. A.*, **108**, 17991–17996.

85. Cui,H., Hu,Y., Guo,D., Zhang,A., Gu,Y., Zhang,S., Zhao,C., Gong,P., Shen,X., Li,Y., *et al.* (2018) DNA methyltransferase 3A isoform b contributes to repressing E-cadherin through cooperation of DNA methylation and H3K27/H3K9 methylation in EMT-related metastasis of gastric cancer. *Oncogene*, **37**, 4358–4371.

86. Xie,M., Zhang,L., He,C., Xu,F., Liu,J., Hu,Z., Zhao,L. and Tian,Y. (2012) Activation of Notch-1 enhances epithelial-mesenchymal transition in gefitinib-acquired resistant lung cancer cells. *J. Cell. Biochem.*, **113**, 1501–1513.

87. Liu,L., Chen,X., Wang,Y., Qu,Z., Lu,Q., Zhao,J., Yan,X., Zhang,H. and Zhou,Y. (2014) Notch3 is important for TGF-β-induced epithelial-mesenchymal transition in non-small cell lung cancer bone metastasis by regulating ZEB-1. *Cancer Gene Ther.*, **21**, 364–372.

88. Xiong,L., Wen,Y., Miao,X. and Yang,Z. (2014) NT5E and FcGBP as key regulators of TGF-1-induced epithelial-mesenchymal transition (EMT) are associated with tumor progression and survival of patients with gallbladder cancer. *Cell Tissue Res.*, **355**, 365–374.

89. Gilkes,D.M., Bajpai,S., Chaturvedi,P., Wirtz,D. and Semenza,G.L. (2013) Hypoxia-inducible factor 1 (HIF-1) promotes extracellular matrix remodeling under hypoxic conditions by inducing P4HA1, P4HA2, and PLOD2 expression in fibroblasts. *J. Biol. Chem.*, **288**, 10819–10829.

90. Yang,J., Shultz,R.W., Mars,W.M., Wegner,R.E., Li,Y., Dai,C., Nejak,K. and Liu,Y. (2002) Disruption of tissue-type plasminogen activator gene in mice reduces renal interstitial fibrosis in obstructive nephropathy. *J. Clin. Invest.*, **110**, 1525–1538.

91. Risolino,M., Mandia,N., Iavarone,F., Dardaei,L., Longobardi,E., Fernandez,S., Talotta,F., Bianchi,F., Pisati,F., Spaggiari,L., *et al.* (2014) Transcription factor PREP1 induces EMT and metastasis by controlling the TGF-β-SMAD3 pathway in non-small cell lung adenocarcinoma. *Proc. Natl. Acad. Sci. U. S. A.*, **111**, E3775-84.

92. Mirza,A., Foster,L., Valentine,H., Welch,I., West,C.M. and Pritchard,S. (2014) Investigation of the epithelial to mesenchymal transition markers S100A4, vimentin and Snail1 in gastroesophageal junction tumors. *Dis. esophagus Off. J. Int. Soc. Dis. Esophagus*, **27**, 485–492.

93. Zhang,J., Luo,A., Huang,F., Gong,T. and Liu,Z. (2020) SERPINE2 promotes esophageal squamous cell carcinoma metastasis by activating BMP4. *Cancer Lett.*, **469**, 390–398.

94. Fenouille,N., Tichet,M., Dufies,M., Pottier,A., Mogha,A., Soo,J.K., Rocchi,S., Mallavialle,A., Galibert,M.-D., Khammari,A., *et al.* (2012) The epithelial-mesenchymal transition (EMT)

regulatory factor SLUG (SNAI2) is a downstream target of SPARC and AKT in promoting melanoma cell invasion. *PLoS One*, **7**, e40378.

95. Miao,L., Wang,Y., Xia,H., Yao,C., Cai,H. and Song,Y. (2013) SPOCK1 is a novel transforming growth factor-β target gene that regulates lung cancer cell epithelial-mesenchymal transition. *Biochem. Biophys. Res. Commun.*, **440**, 792–797.

96. Park,M.Y., Kim,K.R., Park,H.S., Park,B.-H., Choi,H.N., Jang,K.Y., Chung,M.J., Kang,M.J., Lee,D.G. and Moon,W.S. (2007) Expression of the serum response factor in hepatocellular carcinoma: implications for epithelial-mesenchymal transition. *Int. J. Oncol.*, **31**, 1309–1315.

97. Pardali,E., Sanchez-Duffhues,G., Gomez-Puerto,M.C. and Ten Dijke,P. (2017) TGF-β-Induced Endothelial-Mesenchymal Transition in Fibrotic Diseases. *Int. J. Mol. Sci.*, **18**.

98. Medici,D., Potenta,S. and Kalluri,R. (2011) Transforming growth factor-β2 promotes Snail-mediated endothelial-mesenchymal transition through convergence of Smad-dependent and Smad-independent signalling. *Biochem. J.*, **437**, 515–520.

99. Kim,W., Kim,E., Lee,S., Kim,D., Chun,J., Park,K.H., Youn,H. and Youn,B. (2016) TFAP2C-mediated upregulation of TGFBR1 promotes lung tumorigenesis and epithelial-mesenchymal transition. *Exp. Mol. Med.*, **48**, e273.

100. Pino,M.S., Kikuchi,H., Zeng,M., Herraiz,M.-T., Sperduti,I., Berger,D., Park,D.-Y., Iafrate,A.J., Zukerberg,L.R. and Chung,D.C. (2010) Epithelial to mesenchymal transition is impaired in colon cancer cells with microsatellite instability. *Gastroenterology*, **138**, 1406–1417.

101. Gervasi,M., Bianchi-Smiraglia,A., Cummings,M., Zheng,Q., Wang,D., Liu,S. and Bakin,A. V (2012) JunB contributes to Id2 repression and the epithelial-mesenchymal transition in response to transforming growth factor-β. *J. Cell Biol.*, **196**, 589–603.

102. Inai,K., Burnside,J.L., Hoffman,S., Toole,B.P. and Sugi,Y. (2013) BMP-2 induces versican and hyaluronan that contribute to post-EMT AV cushion cell migration. *PLoS One*, **8**, e77593.

103. Mendez,M.G., Kojima,S.-I. and Goldman,R.D. (2010) Vimentin induces changes in cell shape, motility, and adhesion during the epithelial to mesenchymal transition. *FASEB J. Off. Publ. Fed. Am. Soc. Exp. Biol.*, **24**, 1838–1851.

104. Wang,B., Tang,Z., Gong,H., Zhu,L. and Liu,X. (2017) Wnt5a promotes epithelial-to-mesenchymal transition and metastasis in non-small-cell lung cancer. *Biosci. Rep.*, **37**.

105. Wellner,U., Schubert,J., Burk,U.C., Schmalhofer,O., Zhu,F., Sonntag,A., Waldvogel,B., Vannier,C., Darling,D., zur Hausen,A., *et al.* (2009) The EMT-activator ZEB1 promotes tumorigenicity by repressing stemness-inhibiting microRNAs. *Nat. Cell Biol.*, **11**, 1487–1495.

106. Vandewalle,C., Comijn,J., De Craene,B., Vermassen,P., Bruyneel,E., Andersen,H., Tulchinsky,E., Van Roy,F. and Berx,G. (2005) SIP1/ZEB2 induces EMT by repressing genes of different epithelial cell-cell junctions. *Nucleic Acids Res.*, **33**, 6566–6578.

107. Giambra,V., Jenkins,C.R., Wang,H., Lam,S.H., Shevchuk,O.O., Nemirovsky,O., Wai,C., Gusscott,S., Chiang,M.Y., Aster,J.C., *et al.* (2012) NOTCH1 promotes T cell leukemia-initiating activity by RUNX-mediated regulation of PKC-θ and reactive oxygen species. *Nat. Med.*, **18**, 1693–1698.
